# 3D‐AJP: Fabrication of Advanced Microarchitected Multimaterial Ceramic Structures via Binder‐Free and Auxiliary‐Free Aerosol Jet 3D Nanoprinting

**DOI:** 10.1002/advs.202405334

**Published:** 2025-02-07

**Authors:** Chunshan Hu, Sanjida Jahan, Bin Yuan, Rahul Panat

**Affiliations:** ^1^ Department of Mechanical Engineering Carnegie Mellon University Pittsburgh PA 15213 USA; ^2^ Robotics Institute Carnegie Mellon University Pittsburgh PA 15213 USA; ^3^ Manufacturing Futures Institute Carnegie Mellon University Pittsburgh PA 15213 USA

**Keywords:** additive manufacturing, aerosol jet 3D printing, ceramic manufacturing, microarchitected materials, multimaterial manufacturing

## Abstract

Manufacturing of ceramics is challenging due to their low toughness and high hardness. Additive Manufacturing (AM) has been explored to create complex ceramic structures, but current techniques face a tradeoff between precisely controlled feature sizes and high shrinkage at the microscales. Here, we introduce 3D‐AJP, a novel freeform ceramic fabrication method that enables highly complex microscale 3D ceramic architectures—such as micropillars, spirals, and lattices—with minimal shrinkage and no auxiliary support. Using a near‐binder‐free nanoparticle ink in an Aerosol Jet (AJ) 3D printer, our approach precisely controls feature sizes down to 20 µm with aspect ratios up to 30:1. The resulting structures exhibit exceptionally low linear shrinkage of 2‐6% upon sintering, spanning five orders of magnitude in length scale. Bi‐material 3D architectures (zinc oxide/zirconia, zinc oxide/titania, titania/zirconia) and hybrid ceramics further demonstrate the technique’s versatility. We showcase two key applications. First, 3D ceramic photocatalysts improve water purification performance, achieving a 400% increase in photocatalytic efficiency compared to bulk ceramics. Second, we develop a highly sensitive Her2 biomarker sensor for breast cancer detection, achieving a 22‐second response time and a record‐low detection limit of 0.0193 fm. Our technique will lead to high‐performance sensing, filtration, microelectronics packaging, catalysis, and tissue regeneration technologies.

## Graphical abstract

A new paradigm in manufacturing microarchitected multimaterial 3D ceramics is demonstrated. Precise ceramic microlattices, spirals, and microwalls with ultralow shrinkage are achieved via droplet‐based printing without any auxiliary support. ZnO ceramic microwalls via this technique are demonstrated as electrodes for breast cancer biomarker detection exhibiting a record low limit of detection of 0.093 fm and a detection time of 20–30 s.

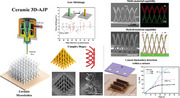

## 1 Introduction

Advanced ceramics are used in several engineering applications because of their wear resistance, thermal stability, thermal insulation, high stiffness, and biocompatibility.^[^ ^1^ ^]^ Amongst these, 3D ceramics with microscale architectures are believed to be the key in developing a number of emerging engineering systems such as lightweight high‐strength structural materials,^[^ ^2^ ^]^ thermal and environmental barrier coatings (TBC/EBC),^[^ ^3^ ^]^ biomedical implants,^[^ ^4^ ^]^ catalysts,^[^ ^5^ ^]^ sensors,^[^ ^6^ ^]^ and microfilters. The manufacture of defect‐free near‐net‐shape porous 3D microarchitected ceramics with precisely controlled architectures, however, is not straightforward due to the lack of availability of reliable ceramic micromachining techniques. Although conventional manufacturing processes such as replica molding,^[^ ^7^ ^]^ sacrificial template,^[^ ^8^ ^]^ direct foaming,^[^ ^9^ ^]^ freeze casting,^[^ ^10^ ^]^ injection molding,^[^ ^11^ ^]^ gel casting,^[^ ^12^ ^]^ and slip casting^[^ ^13^ ^]^ are capable of fabricating 3D ceramic materials either by dry forming or wet forming, their ability to generate features at the micrometer scale is limited due to multistep processes that lead to the lack of control over the dimensions. In addition, the reliance on molds and dies hinders the customizability of the manufacturing process to produce on‐demand intricate features.

Recent advances in additive manufacturing (AM), colloquially known as 3D printing, have led to several possibilities of fabricating different material geometries and combinations.^[^ ^14^ ^]^ These techniques are divided into two primary types, namely, “multistep” and “single‐step” processes. The former includes inkjet printing (IJP),^[^ ^15^ ^]^ binder jetting (BJ),^[^ ^16^ ^]^ fused deposition modeling (FDM),^[^ ^17^ ^]^ and stereolithography (SLA),^[^ ^18^ ^]^ and the latter includes selective laser sintering (SLS)^[^ ^19^ ^]^ and laser engineered net shaping (LENS).^[^ ^20^ ^]^ For multistep processes, severe linear shrinkage (15–43%) and/or defects during the postprinting processing are often observed due to the removal of the additives in the ink (e.g., binders and additives that are needed as supportive and sacrificial materials) or decomposition of the preceramic polymer (i.e., burn out of the polymer components during sintering/pyrolysis). In addition, binders in many cases raise safety concerns due to hazardous handling requirements. For single‐step processes, although near‐net‐shape structures can be fabricated, the limitation on the choices of materials and resolution makes it challenging to make parts with microscale features. In addition, for most of the current AM processes, it is challenging to realize 3D hierarchical porous ceramic structures with high printing resolution and fabrication speed due to processes such as de‐binding, sintering/pyrolysis, and surface finishing. A gap thus exists in the fabrication of precise near‐net‐shape hierarchically porous complex 3D ceramic microarchitectures with minimal volume shrinkage and shape distortion.

Aerosol jet (AJ) nanoprinting is a jetting‐based AM technique that can print nanomaterials at micrometer feature sizes.^[^ ^21^ ^]^ Our earlier work demonstrated complex 3D metal architectures where the struts were constructed at a length scale of 10 µm with controlled mechanical properties.^[^ ^22^ ^]^ The printed structures were used in several exciting applications such as ultrafast COVID‐19 antibody and antigen detection,^[^ ^23^ ^]^ mechanically tunable lightweight high‐strength structural materials,^[^ ^22b^ ^]^ ultra‐high‐density brain–computer interfaces that can record neuronal signals from throughout the 3D volume of the brain,^[^ ^24^ ^]^ high specific capacity lithium‐ion batteries,^[^ ^25^ ^]^ and highly conductive flexible electronic circuits.^[^ ^26^ ^]^ Combining multimaterials in a single structure at microscales is challenging. AJ printing has been used to combine multiple materials in 2D films to fabricate alloys with variable compositions.^[^ ^14c^ ^]^ This work, however, is confined to planar films. Stacked metallic multimaterial structures (e.g., Cu/Co) via a 2‐photon polymerization (2PP) followed by hydrogel infusion has been demonstrated.^[^ ^27^ ^]^ This technique, however, has multiple process steps, and the structure formation is slow for practical applications. AJP of 3D structures has also been demonstrated for Indium tin oxide (ITO) micropillars^[^ ^28^ ^]^ for photosynthesis and graphene for moisture sensors.^[^ ^29^ ^]^ The above works, however, do not cover: i) traditional structural and functional ceramics such as zirconia, alumina (Al_2_O_3_), or titania, where complex shapes can be made only using AM, but have a significant shrinkage; ii) complex ceramic structures such as 3D microlattices and spirals at a scale required for surface‐sensitive applications such that they bridge the length‐scale gap between nanoscale ceramic films/sieves and macroscale 3D structures made by binder jetting; and iii) multimaterial/hybrid ceramic systems. Current work is thus motivated by filling the gaps discussed above and the desire to extend the extant ceramic fabrication capability by using binder‐free ceramic nanoparticle inks via AJ nanoprinting.

We expect 3D microarchitected ceramics to open up a hitherto unexplored fabrication space which is critical to several device applications such as biosensing^[^ ^30^ ^]^ and water purification.^[^ ^20^, ^31^ ^]^ For example, oxide nanoparticles are well known for their superior biosensing properties due to high surface area, chemical stability, biocompatibility, and an ability to immobilize biomolecules on surfaces by isoelectric point (IEP)‐driven electrostatic absorption.^[^ ^30a^ ^]^ The current implementations of ceramic biosensors, however, are limited to 2D planar nanostructures.^[^ ^32^ ^]^ Extending these to 3D architecture would enable significantly enhanced surface interactions for sensing. Furthermore, surface‐sensitive applications such as water purification can greatly benefit from hierarchical 3D porous structures, which provide enhanced surface area and tailored flow paths for effective pollutant removal.

In this study, we demonstrate novel ceramic structures such as complex 3D microarchitectures (freeform microspirals, microlattices, micropyramids, etc.) via AJ 3D nanoprinting (we call the process “3D‐AJP”) with near zero shrinkage and a minimum feature size of 20 µm. Hierarchical porosity with pore sizes ranging from tens of nanometer to a millimeter (spanning over five magnitudes of length scales) is designed and engineered through acombination of AJ‐nanoparticle‐based support‐free printing as well as controlled postprocessing. Taking advantage of the controlled porosity at various length scales as well as the high surface‐area‐to‐volume ratio, we show excellent photocatalytic performance of ZnO microlattices for the degradation of methylene orange dye in wastewater. When compared with bulk ZnO, our structures exhibit three times the degradation speed per unit area under irradiation, demonstrating a significant improvement and potential in photocatalysis applications. A breast cancer detection platform with ultrahigh sensitivity is also demonstrated, leveraging the enhanced surface area provided by our ceramic 3D‐AJP technology.

## 2 Results and Discussion

### 2.1 3D‐AJP Using Binder‐Free ZnO Nanoparticle Ink

**Figure 1:**
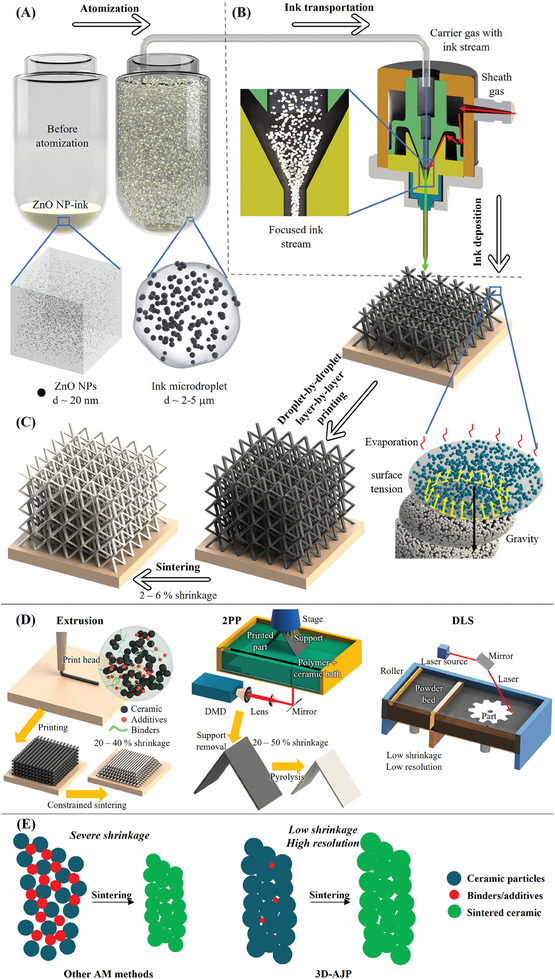
Schematic of 3D‐AJP: aerosol Jet 3D nanoprinting of intricate 3D ceramic microarchitectures with near‐zero shrinkage. A) ZnO nanoparticle‐based ink atomized by the ultrasonic atomizer of an AJ nanoparticle‐based printer, with the zoomed‐in images showing the nanoparticles (diameter ≈ 20 nm) in the ink vial and a single droplet. B) The aerosol (mist) being transferred to the deposition head through the tubing system using an inert gas. The zoomed‐in image shows the loosely packed ink stream focused by the sheath gas onto the substrate. C) Rapid droplet‐by‐droplet, freeform printing without auxiliary support. The zoomed‐in image illustrates the mechanism of building inclined structures without auxiliary support. Minimum shrinkage is observed after the sintering demonstrating the near‐net fabrication. D) Ceramic fabrication by AM techniques such as extrusion printing, 2PP, and DLS. E) Schematics showing the use of precursor inks with high binder content (left) and minimal binder content (right) for ceramic manufacturing.

**Figure** **1**A–C shows the schematics of the 3D‐AJP process developed in this work, while Figure 1D shows schematics of three (other) representative additive techniques, namely, extrusion, 2PP, and SLS, for the fabrication of 3D ceramic structures. For extrusion printing, a slurry containing ceramic particles, binders, and additives is extruded through a nozzle that typically involves high stress and depends on the rheological properties of the ink, such as viscosity and shear thinning. The fraction of polymers in the slurry is typically high, leading to a significant shrinkage and issues such as cracks during sintering and highly anisotropic mechanical properties due to print orientation. In addition, the resolution of the extrusion printing is limited by the nozzle diameter, which is typically larger than 100 µm to ensure the flowability of the ink. Another multistep AM technique, 2PP, offers ultrahigh resolution with minimum feature sizes in the range of tens to hundreds of nanometers. However, this process is slow for practical applications and often suffers from a high shrinkage (20–50%) due to the mandatory addition of polymers required by the polymerization process.^[^ ^33^ ^]^ Single‐step AM techniques such as digital light synthesis (DLS) have the capability to melt/sinter the powders of various materials while printing and require no further sintering; thus, they possess low shrinkage. However, the feature sizes are limited by the sizes of the powders and the laser spot, making it impossible to fabricate fine features smaller than 150–200 µm.

The schematic of the ceramic 3D‐AJP process developed in this work to achieve intricate microarchitectures with near‐zero shrinkage is shown in Figure 1A (see the “Experimental Section” for details of the inks and the process). The AJ nanoparticle‐based printer is equipped with two power adjustable high‐frequency (MHz) ultrasonic atomizers that can induce a capillary wave on the ink surface, which helps generate microdroplets with different densities and viscosities in relatively well‐defined size distributions (see the zoomed‐in schematic of Figure 1A). The microdroplets containing ceramic nanoparticles are transported to the deposition head through a carrier gas, as shown in Figure 1B. A sheath gas focuses the ink droplets from the mist into a high‐speed ink stream with diameters up to 10–15 times smaller than the inner diameter of the nozzle (i.e., ≈10–15 µm if the nozzle diameter is around 150 µm). The accuracy of the platen is ±1 µm at 3σ. AJ nanoprinting “does not depend” upon the rheological properties of the ink. The microdroplets deposited on the substrate are expected to lose the solvent rapidly due to the high surface‐area‐to‐volume ratio once in contact with the already formed structure or substrate. Note that as the droplet diameter/size decreases, the surface forces decrease at a slower rate than the inertia of the droplet, leading to “sticking” of the droplet with the structure already formed beneath. This property can be highly useful in forming 3D structures “without” any auxiliary support.^[^ ^22a^ ^]^ The substrate is often heated to speed up the evaporation process (and modulates the dimension of the printed structure as will be seen later). The zoomed‐in schematic of Figure 1C shows the process of rapid evaporation of the solvent in the ink, leaving dried solid nanoparticles.

To ensure low shrinkage after sintering, two critical conditions must be met. First, the nanoparticles must be closely packed after printing. Second, the binder/additives in the nanoparticle ink must be minimal to ensure the lowest amount of weight/volume lost during sintering. Commercially available inks often consist of binders/additives that hold and draw nanoparticles together and form a physically and chemically stable dispersion. However, binders are mostly polymers whose melting/evaporation point is much higher than the evaporation temperature of the solvent (also typically higher than the platen temperature of the AJ printer). This decomposition process under higher temperature sintering leads to shrinkage and can be as high as 20–50% if the binder content is high.^[^ ^34^ ^]^ The schematics in Figure 1E show this process with and without significant amounts of binders.

Ceramic nanoparticles are typically more stable than metallic nanoparticles due to their chemical inertness.^[^ ^35^ ^]^ As a result, lower fractions of binder/additive are needed to stabilize the nanoparticle inks.^[^ ^36^ ^]^ We obtained commercially available ZnO and ZrO_2_ nanoparticle dispersions with minimal binders for this study (see the “Experimental Section” for details). The ultralow binder/additive concentration in the ZnO nanoparticle‐based ink was confirmed by thermal gravimetric analysis (TGA) in air at a heating rate of 10 °C min^−1^, as shown in Figure S1A (Supporting Information). Note that initially, the weight of the ink decreased rapidly with increasing temperature. At *T* = 90 °C, the weight dropped to 24.38% of the original, which slowly decreased to 24.19% at 600 °C indicating that the weight percentage of binders and other additives in the ink that may cause shrinkage after sintering was <0.19%, and the solvent used for dispersion was likely removed at *T* = 90 °C. Since the binder/additive content is not revealed by the vendor, we assume that the binder used in ZnO nanoparticle ink has a density in the range of 1–2 g cm^−3^, which is typical for common binders. Thus, we can estimate the volume percent of the binders in the printed green structure. ZnO has a density of 5.61 g cm^−3^, giving 0.19 wt% or 2.16– 4.22% vol% of binder in the printed 3D structure. Considering that the ink would be atomized and broken into droplets of aerosol mist during printing, a high surface‐area‐to‐volume ratio for the solvent is expected (e.g., for 1 µm diameter droplet, the surface‐area‐to‐volume ratio is 3 × 10^6^ vs 3 × 10^3^ m^−1^ for a 1 mm diameter droplet). Thus, the solvent could be dried/removed once the ink was deposited, leaving the green part consisting of mostly dried ZnO nanoparticles. Although the amount is significantly low, the weight percentage of the binder/additives/surfactants is ≈0.86%, which is essential to keep the nanoparticles stable in the solvent without agglomeration. Note that the weight percentage of the binder/additives/surfactants in other commercially available AJ printable metallic (silver (Ag) and gold (Au)) nanoparticle inks is ≈7–10% from our measurement via TGA (Figure S2, Supporting Information). Transmission electron microscopy (TEM) was performed on the ink, and the ZnO nanoparticles are shown in Figure S1B (Supporting Information). Note that the particle sizes are in the range of 35 ± 7 nm in diameter, which is within the recommended range of particle sizes by the manufacturer and suitable for AJ printing.

### 2.2 AJ Printability Study for Planar and 3D Structures

We first evaluated the 2D printability of the ink. Figure S1C (Supporting Information) shows the scanning electron microscopy (SEM) images of the AJ nanoprinted 2D traces after printing on an alumina substrate at 50 °C at a speed of 1 mm s^−1^. The sheath gas flow rate was set to be 45–55 sccm, while the carrier gas flow rate was set to be 17–23 sccm. At low carrier gas flow rates (<19 sccm), rough edges/surfaces and loosely packed structures were observed, indicating an underdeposited condition. With increasing carrier gas flow rate (19–21 sccm), the edges of the printed traces are clearly defined, as shown in the green zone, indicating an optimized printing condition. Images marked by red triangles in Figure S1C (Supporting Information) indicate material accumulation in the lines, which is an undesirable condition. Figure S1D (Supporting Information) shows a zoomed‐in image of a trace in the green zone. Prior to the fabrication of the 3D structures, we characterized the influence of the platen temperature on the “flow” of the printed material. A higher platen temperature can speed up the evaporation of the solvent, which is essential for the formation of the 3D structures. Figure S1E (Supporting Information) shows the diameter of the deposited droplets (spread after impact with the substrate) as a function of the platen temperature for the same printing program. Clearly, the droplet size can be modulated by the platen temperature via the control of the flow of the material during drying.

We next explored the possibility of printing 3D microscale structures with the complexity shown in the schematic of Figure 1C. Specifically, the hypothesis was that the droplets from the aerosol in the AJ nanoprinting process can be “stacked” on one another via a combination of rapid evaporation and surface forces to form 3D microarchitectures such as microlattices. The low binder content will then lead to minimal distortion after sintering to create the structures indicated in Figure 1C. To set the stage for complex structures, we evaluated the fabrication of inclined micropillars—the building block for a complex 3D microlattice—using ZnO ink. A CAD program was developed where the distance between the droplets, *δ*, was successively increased to achieve ZnO micropillars with increasingly acute angles with the horizontal plane.

**Figure 2:**
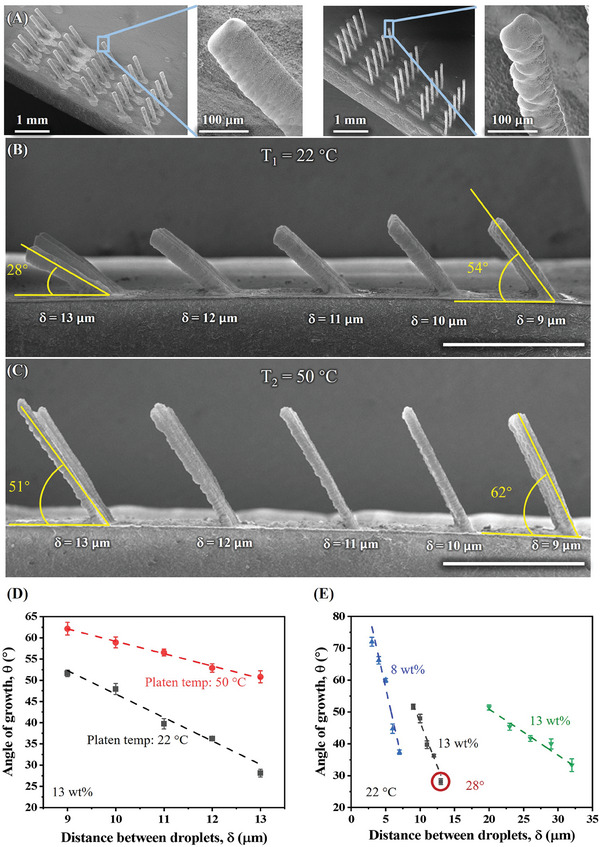
Fabrication of inclined ZnO micropillars via 3D‐AJP. A) Perspective SEM images of micropillars and zoomed‐in images showing their surface roughness for printing on a platen at room temperature (left) and at 50 °C (right). For each micropillar, the distance, *δ*, between the successive droplets is increased from right to left. B,C) Side‐view SEM images of micropillars in panel (A) showing the values of *δ* being used for the particular sets of micropillars. The angle of the printed micropillars with the horizontal, i.e., angle of growth, is also indicated in some cases. Scale bars: 1 mm. D) Angle of growth as a function of distance between droplets, *δ*, on alumina substrates for printing at both, room temperature and 50 °C. E) Effect of *δ* at different ink concentrations (8%, 13%, and 20% in weight) for a given printing temperature on the angle of growth of the micropillars.

**Figure 3:**
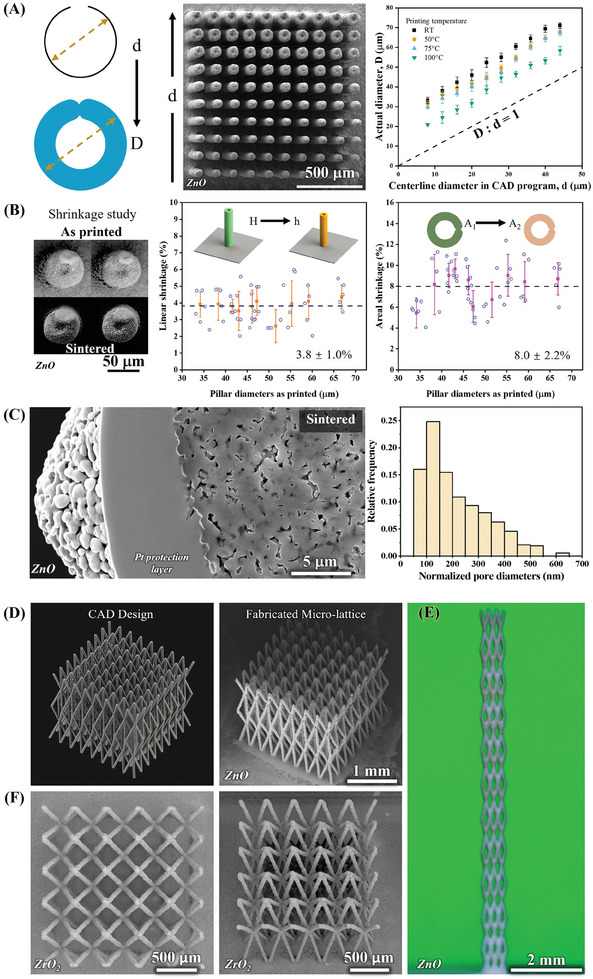
Printability of 3D ceramic microarchitectures via 3D‐AJP, along with the measured shrinkage. A) Schematic of printing trajectory in CAD program versus actual diameter of printed micropillars. The realized diameter of the micropillars is expected to be a sum of “*d*,” the ink stream diameter + any flow prior to solidification (left schematic). The middle image shows top view SEM of printed micropillars with increasing “*d*” from bottom to top. Ten micropillars were printed for each “*d*” for statistical analysis. The relationship between actual printed diameter and “*d*” at different printing temperatures is plotted on the right showing a consistent increase for printing at different temperatures. B) Optical images of ZnO micropillars before and after sintering (left), linear shrinkage (middle), and areal shrinkage (right) for different micropillar diameters. C) SEM of focused ion beam (FIB) section of one strut from the microlattice (left), and corresponding normalized pore size distribution (right) showing an average pore diameter of 214 nm. D) CAD design versus fabricated ZnO microlattice realized by our 3D‐AJP technique. E) Optical image of high aspect ratio microlattice (a height of 1 cm and a width of 600 µm). F) Top (left) and tilted SEM view (right) of microlattice of a different ceramic, namely, ZrO_2_.

**Figure** **2**A shows the prospective SEM images of ZnO micropillars printed at platen temperatures of 22 °C (left) and 50 °C (right) via the 3D‐AJP method. All other printing parameters were kept identical between the two cases. No auxiliary support was used during printing. For each case, *δ* was increased from 9 to 13 µm with the centerline diameter in the CAD program being kept at 20 µm. Our technique forms well‐defined ZnO micropillar structures, as evidenced by Figure 2A. In addition, the process is controllable with increasingly acute angles to the horizontal with increasing distance between the droplets (*δ*) during printing. The aspect ratio of the inclined micropillar printed at 50 °C platen temperature is higher than that printed at room temperature, possibly due to the faster rate of evaporation of the solvent. The insets in Figure 2A show an uneven surface for the micropillars printed at 50 °C, as can be explained by the likely faster evaporation of solvents expected at that temperature, which would prevent surface‐tension‐driven smoothing during the evaporation of the solvents. Figure 2B,C shows side‐view SEM images of the micropillars shown in **Figure** **3**A. The angle of the micropillars with the horizontals in Figure 2B decreased from 54° to 28° for a *δ* value of 9–13 µm, respectively. The resulting diameter of the micropillars is about 96 µm. Figure 2C shows similar data for printing at 50 °C, where the angle of the ceramic micropillar with the horizontal decreased from 62° to 51° for a *δ* value of 9–13 µm, respectively. The diameter of the micropillars in Figure 2C is reduced to about 75 µm. The difference in the diameter and angle observed in Figure 2B,C is likely a function of the evaporation kinetics of the droplets. In fact, the diameter of the printed micropillars is expected to be the sum of the diameter of the printed material and the “settling” of the droplets on the previously formed structure prior to complete evaporation of the solvents. Note that the ink composition and printing conditions used to build the structures in Figure 2A–C are identical except for the platen temperature. The slower evaporation at room temperature for the structures in Figure 2B is expected to allow more time for the droplets to settle and spread, leading to a larger micropillar diameter. The same kinetics will also lead to more acute angles for the pillars as gravity will have more time to act prior to solvent evaporation.

The total volume of material in a micropillar in Figure 2B was comparable to that in Figure 2C, as shown in Table S1 (Supporting Information). The small difference observed in the two cases can be attributed to the excess overspray of droplets, as seen in Figure 2B. The angle of growth as a function of *δ* for the 3D ceramic micropillars in Figure 2A–C is given in Figure 2D. Excellent process control is evident by the standard deviation being within ±2.5% of the mean. To enable the printing of micropillars with increasingly acute angles, one possibility is to continue to increase *δ*. However, this option is limited because when *δ* was increased to ≥ 14 µm for the micropillars in Figure 2B,C, most of the droplets fell onto the substrate and inclined micropillars could not be built. Furthermore, for a given printing diameter, the minimum achievable angle is limited by the printing temperature. Ink concentration is another factor that can affect the critical angle of growth as the evaporation time is expected to be a function of the solid loading (with higher nanoparticle loading leading to faster evaporation). Figure 2E shows a comparison of angles of growth of micropillars for inks with different nanoparticle loadings (8%, 13%, and 20%); all printed at room temperature. The minimum critical angles for these cases were 36°, 28°, and 33°, respectively. For angles <28°, substrate tilting via robotic controls can be utilized to realize such micropillars. This type of instrumentation is currently under development by the authors and can lead to a rich set of results via robotics and control for 3D micromanufacturing, but is beyond the scope of this paper. From the results above, it is concluded that both the platen temperature and ink composition affect the printing outputs, and the micropillar dimensions printed for a given condition are highly predictable and repeatable.

Next, we measured the diameter of the printed structures as a function of the increasing diameter of the centerline of the circular path in the CAD program used for the AJ printer at different platen temperatures. As seen in Figure 3A, the printed diameter is higher than the centerline diameter of the CAD program for all the cases, with consistent (and hence predictable) difference. For printing at 100 °C, the printed diameter is about 12 µm higher than the centerline, indicating that the printed line width is close to that value. This is consistent with the assumption that the diameter of the printed line is ≈1/10^th^ of the nozzle diameter. For printing at lower temperatures (75, 50, and 25 °C), the diameter of the printed micropillar successively increased for a given *δ* compared to that at 100 °C due to increasing time available for the ink to flow before complete evaporation of the solvents (top view of the micropillars printed at 50 °C is shown in the SEM image of Figure 3A). The graph in Figure 3A indicates good predictability and controllability of our printing process since the diameter increased linearly with the *d* and the error bars are within ±2.5–5% of the mean. Note that the mean and error bars are calculated using 10 micropillars per diameter per platen temperature (a total of 400 data points being used for the plot in Figure 3A). These results demonstrate that AJ printing can be used to fabricate micropillars with well‐defined dimensions.

The micropillar structures mentioned above were all “green,” i.e., the nanoparticles were in an unsintered state. Next, we studied the shrinkage of the micropillars upon sintering. It is well established that in nanoparticle‐based colloidal systems, the packing density (≈64– 74%, depending on packing arrangements and particle size distribution) primarily constrains low shrinkage. However, for our demonstrated applications, achieving high‐density sintering is not always necessary. In fact, for applications such as photocatalysis, biomolecule sensing, and microelectronics packaging, functional performance of ceramics can be excellent without fully densifying. ZnO micropillars similar to those in Figures 2A and 3A were sintered at 950 °C for 6 h (see the “Experimental Section”). After sintering, the diameters and heights of the printed pillars were measured using SEM and a high‐magnification optical camera (FL3‐GE‐13S2C, Point Grey, LA, USA), respectively. Five measurements were performed on ten sets of ZnO micropillars with diameters ranging from 33 to 67 µm. Representative SEM images before and after sintering, along with the linear and areal shrinkage (3.8 ± 1.0% and 8.0 ± 2.2%, respectively) are shown in Figure 3B. With the area and shrinkage data for each pillar, we also calculated that volume shrinkage after sintering to be 11.5 ± 2.4% (Figure S3, Supporting Information). This shrinkage is significantly lower than that with other AM techniques mentioned in the “Introduction” section (see **Table** **1** for comparison). We then used focused ion beam (FIB) sectioning to examine the cross section of microlattice truss members under as printed and sintered conditions (Figures 3C and S4A, Supporting Information). The image in Figure 3C shows particles merged upon sintering with low internal porosity (ImageJ analysis in Figure S4B of the Supporting Information). A slight decrease in macroporosity was observed from 9.12% ± 0.65% to 8.62% ± 0.63% after sintering, indicating small overall volume change. This observation supports the low shrinkage observed in Figure 3C and is consistent with the low binder content in the ink. The X‐ray diffraction (XRD) images of the AJ‐printed and AJ‐sintered ZnO are shown in Figure S5 (Supporting Information). The diffraction peaks of the sample represent a standard hexagonal ZnO structure, indicating a high‐purity ZnO material, as no other diffraction peaks observed. Note that the measurements were taken at a 3° tilt to eliminate reflection from the alumina substrate.

**Table 1:** Comparison of different ceramic additive manufacturing methods.

Methods	Material	Additives	Feature size	Resolution	Shrinkage	porosity	Reference
Digital light processing‐stereolithography (DLP‐SLA)	ZrO_2_	1.6‐hexanediol diacrylate (HDDA), photomer 4172 (PPTTA), polyethylene glycol (PEG)D, U600 and 1‐octanol	mm–cm	≈200 µm	35.26%	2.86%	[34]
SLA	ZrO_2_	PEGDA‐575 29.75 wt%	mm–cm	250 µm	20–25%	6–9%	[34b]
2PP‐ALD (hollow)	Alumina	Polymer backbone	5–100 µm	50 nm	N/A	N/A	[37]
2PP	ZrO_2_	33 wt%	From 200 nm to 100 µm	100–200 nm	19%	N/A	[33a]
2PP	ZrO_2_	PEGDA 30 wt%	From 800 nm to100 µm	500 nm	40%	N/A	[33b]
Direct ink writing (DIW)	ZrO_2_	polydimethylsiloxane (PDMS) 60–80 wt%	mm–10 cm	300 µm	20%	N/A	[38]
DIW	Al_2_O_3_	Ammonia, polyvinylpyrrolidone (PVP), and oleic acid (OA)	1–10 cm	1 mm	50 wt%	75–85%	[39]
Extrusion	TiO_2_	50 wt%	5 mm to 5cm	500 µm	4.5–6.8%	N/A	[40]
Extrusion	HA	hydroxypropyl methyl cellulose (HPMC 33 wt%	1–5 cm	400 µm	37%	5–45%	[34c]
Extrusion	ZrO_2_, MoSi_2_, Al_2_O_3_	Thermoplastic	1–10 cm	720 µm	50 vol%; 16% linear	3.4–3.6%	[41]
BJ	Al_2_O_3_	Inorganic colloidal binder	mm to 10 cm	mm	14–20%	35–55%	[42]
BJ	Al_2_O_3_	triethylene glycol dimethacrylate (TEGDMA) and Irgacure 184	mm to 6 cm	mm	11%	25–32%	[43]
BJ	ZrB_2_–MoSi_2_	40 wt%	1.5 mm to 10 cm	mm	26%	2–8%	[44]
SLS	Al_2_O_3_–Al	Aluminum	2 mm to 10 cm	200–500 µm	1%	54.5%	[45]
SLS	Y_2_O_3_	N/A	mm to 10 cm	200–300 µm	1–10%	41%	[46]
**3D‐AJP (this work**)	ZnO, ZrO_2_	0.86 wt% binders	10 µm to 1 cm	10 µm	2–5%	9%	

### 2.3 Complex 3D Ceramic Microarchitectures with Hierarchical Length Scales and Multimaterial Capability

To design the complex 3D microarchitectures, commercially available AutoCAD package was used. Figure S6 (Supporting Information) shows a representative design for a lattice having 3×3×5 unit cells. The drawing in AutoCAD is defined by droplet‐by‐droplet distance (*δ*), diameter of the droplet (*d*), number of unit cells in *x‐* and *y*‐directions (*m*), and the number of layers (*n*). These parameters will then determine the physical parameters of the 3D geometry such as pillar diameter (*D*), unit cell height (*h*), number of unit cells in the *z*‐direction (*n_z_*), total structure height (*H*), truss angle (*θ*), overall lateral dimension (*L*). Specifically, we determine *θ* and *h* for the lattice, then based on the results extracted from Figure 2B,D, we calculate the desirable *δ* for a *θ* with required *n* to reach the height *h* based on the layer thickness measured from the same figure. With this information, the *H* and *L* can also be determined by *H* = *h* × *n_z_* and *L* = *δ* × *n* × sin 45° × *m*. Next, we assign “*D*” in the CAD model, for which the “*d*” in AutoCAD program can be determined by the relationships shown in Figure 3A. To verify the fidelity of the printed structures to the 3D CAD geometry, we compared the key parameters such as *H*, *L*, *θ*, and *D*, and the results are shown in **Table** **2**.

**Table 2:** Comparison of 3D CAD design and printed ceramic microlattices.

Feature	Designed CAD dimension	Experimental dimension	Difference [%]
Overall height [mm]	4.8	4.9 ± 0.08	2.1 ± 1.7
Overall length [mm]	2.5	2.51	0.4
Overall width [mm]	2.5	2.52	0.8
Diameter [mm]	56	58 ± 3.5	3.6 ± 6.4
Angle [°]	70	71.9	2.71

Figure 3D shows a CAD drawing of an 8×8×3 microlattice and the corresponding AJ‐printed and AJ‐sintered complex ceramic (ZnO) 3D microarchitecture envisioned in this study. As seen in the CAD image, the ZnO microlattice is faithfully reproduced by our technique. Such complex architectures at this length scale are difficult, if not impossible, to make by any other technique. A larger ceramic structure with microarchitected features at small scales is shown in Figure 3E. This 3 × 3 ZnO microlattice has 22 repeated layers in the spatial (*z*)‐direction and has a total height of about 1 cm. The structural length scale, thus, spans from tens of micrometers for the truss segments of the micropillars to 10 mm. To establish generality of our printing, we show printing of a second ceramic material. Figure 3F shows a microlattice with well‐defined truss members of ZrO_2_ ceramic. It is interesting to know that the microlattice shown in Figure 3D takes about 15 min to print, which is fast compared to other techniques such as 2PP. A video of printing of the microlattice similar to that shown in Figure 3D can be seen in Movie S1 (Supporting Information). In order to quantify the variability in dimensions of the truss members of the 3D microlattices fabricated by our technique, we printed a lattice structure with two radii (Figure S7, Supporting Information). The variability for two truss diameters was within ±6% (Figure S7B, Supporting Information). Such excellent uniformity of the ZnO 3D structures can be attributed to our 3D‐AJP process and to the minimal binder content in the ink.

**Figure 4:**
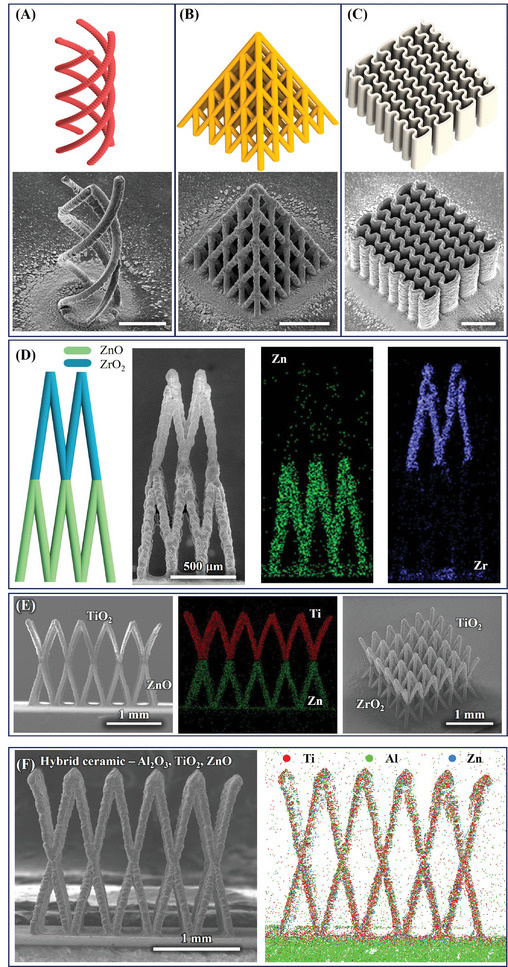
AJ‐nanoprinted complex single‐ and bimaterial ceramic microarchitectures. CAD design (top) and SEM images (bottom) for A) multispiral microarchitecture, B) pyramidal microlattice, and C), wavy microwalls of ZnO. The scale bars are 500 µm. D) Printed bimaterial pyramidal microlattice (left) with energy‐dispersive X‐ray spectroscopy (EDX) showing Zr and Zn on different printed layers (right). E) Additional bimaterial microlattice including TiO_2_ on top and ZnO on bottom with EDX confirming the composition. Another bimaterial structure with TiO_2_ on top and ZrO_2_ on bottom is also shown. F) Hybrid ceramic materials containing Al_2_O_3_, TiO_2_, and ZnO with EDX showing uniformly distributed Ti, Al, and Zn in a single lattice.

Various unusual and visually exciting 3D microarchitectures of ZnO fabricated by 3D‐AJP are shown in **Figure** **4**. A CAD image of four intertwined spirals, each with an aspect ratio of about 30:1, is shown in Figure 4A. The sintered AJ‐printed ZnO spirals are also shown, which mirror the 3D CAD design. Our process thus faithfully replicates the CAD designs. Figure 4B,C shows CAD images of a pyramidal lattice and wavy microwalls, respectively. The corresponding sintered ZnO structures demonstrate excellent reproducibility. Figure S8 (Supporting Information) shows dumbbell‐shaped 3D structures with limited overhangs that are also fabricated using our approach.

We now demonstrate the versatility of 3D‐AJP by showing a multimaterial 3D microarchitecture. Here, we integrate ZnO and ZrO_2_ within the same lattice structure, enabling the fabrication of a micropyramid with different materials in different sections (Figure 4D). The base of the micropyramid is constructed from ZnO while the apex from ZrO_2_ as seen in the CAD image in Figure 4D. A seamless transition between these two materials within the micropyramid is achieved through precise control of the AJ nanoprinting process, allowing for the deposition of each material in predetermined locations without the need for physical barriers or complex postprocessing steps. Figure 4D shows the resulting bimaterial micropyramid structure. To confirm the material composition, EDX analysis was employed, which shows a clear demarcation between the materials—Zn denoted by green and Zr by blue as seen in Figure 4D (right). In addition, we have also demonstrated the two more bimaterial microlattice structure with TiO_2_ printed on top of ZnO (SEM shown in Figure 4E (left) and EDX shown in Figure 4E (middle)) and ZrO_2_ (Figure 4E, right), respectively. This not only demonstrates the ability of our approach to fabricate intricate 3D structures, but also highlights the ability to concurrently manipulate material composition within a unified architecture.

Building on this success, we expanded the capabilities to create multimaterial systems by fabricating hybrid microlattice structures using a single ink containing a homogeneous mixture of ZnO, TiO₂, and ZrO₂ nanoparticles. As seen in Figure 4F, this approach allows for the simultaneous 3D co‐deposition of multiple functional metal oxides within a single microarchitected lattice. The resulting composite microlattice shows a distribution of the three oxides throughout the structure as seen in the SEM and EDX elemental maps shown in Figure 4F. This demonstration highlights the versatility and precision of our technique in realizing hybrid ceramic microarchitectures, paving the way for the development of advanced materials with tailored properties for applications in catalysis, sensing, and photonic devices where the integration of multiple functional materials may be required.

**Figure 5:**
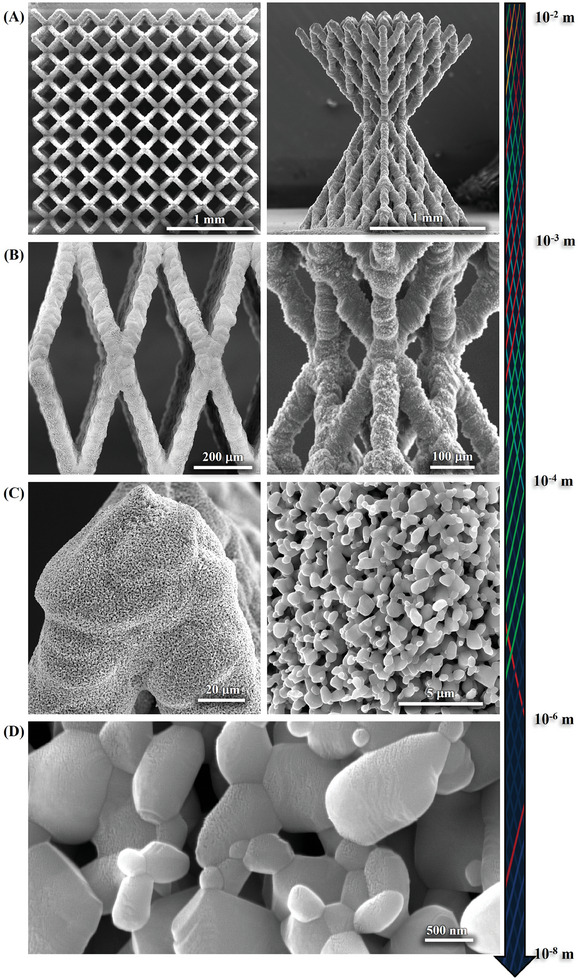
SEM images highlighting hierarchical length scales of ZnO 3D microlattices enabled by our ceramic 3D‐AJP technique. A) SEM images of ZnO microlattices. Left: top view of an 8×8×2 microlattice, and right: front view of a double pyramid with eight layers at a length scale of a millimeter. B) Close‐up SEM images of panel (A) showing the truss members with diameters in the range of 30–80 µm and periodicity of the lattice in the range of 100–350 µm. C) SEM images showing the porosity formed by sintered particles on the surface of the structures in panel (B). D) Close‐up SEM showing nanoscale pores on the surface of the structure in panel (C).

**Figures** **5** and S9 (Supporting Information) highlight the length scale of the 3D microarchitected ceramics fabricated by our technique. The microlattices and “double‐pyramid” structures (with octahedral unit cells) in Figures 5A and S9A (Supporting Information) have an overall dimension of a few millimeters. The diameter of the truss elements of each of these structures (Figures 5B and S9B, Supporting Information) is of the order of 30–80 µm. The periodicity of the lattice is in the range of 100–350 µm. Lastly, sintering of the ZnO nanoparticles can also introduce two levels of pores, as shown in Figures 5C and S9C (Supporting Information) where necking and merging of nanoparticles induced by sintering are evident. These particles create surface pores, ranging from hundreds of nanometers to micrometers, greatly increasing the surface area and roughness.

### 2.4 Application Demonstration: Highly Efficient Photocatalysis for Water Purification

**Figure 6:**
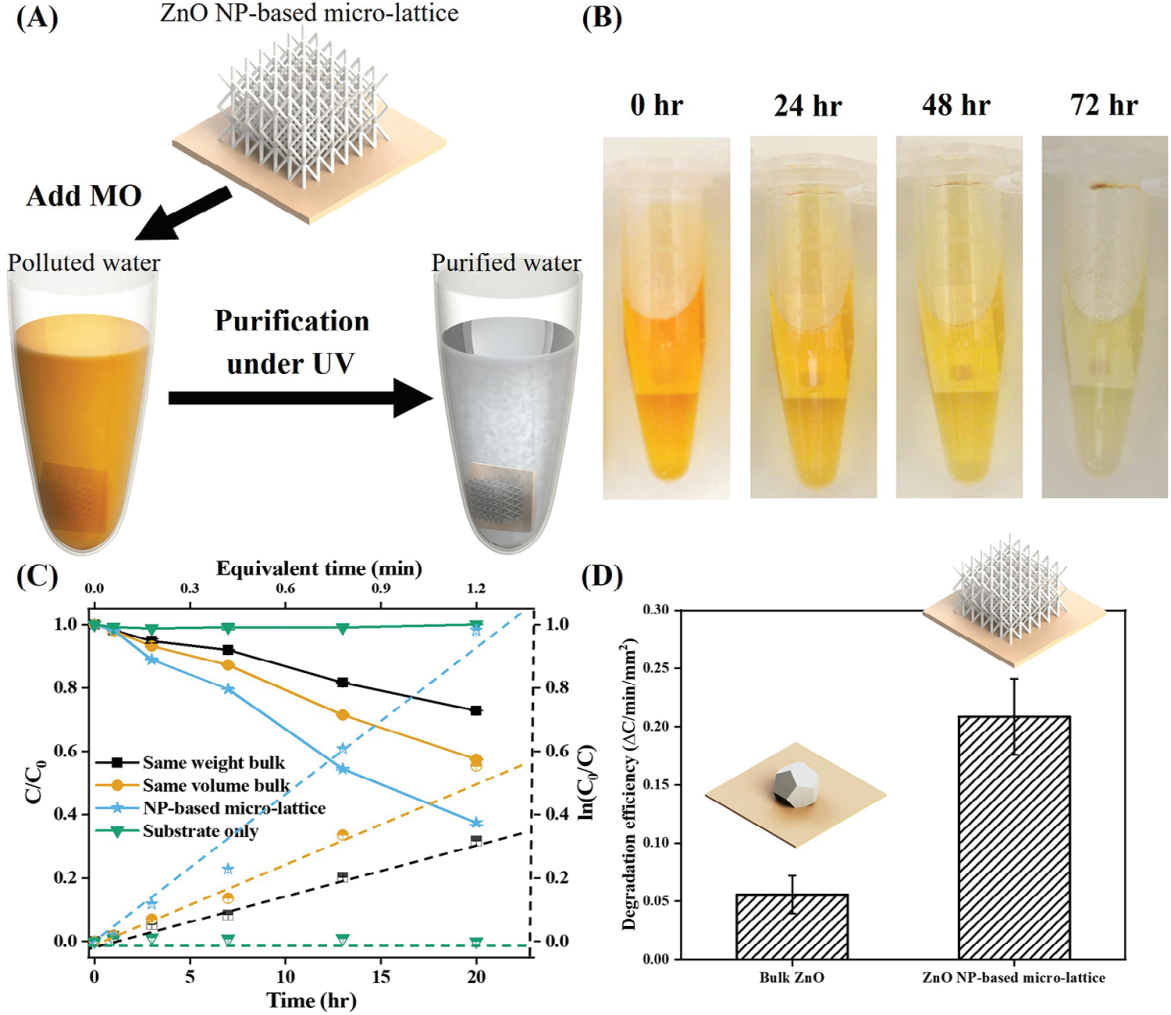
3D ZnO microlattice via 3D‐AJP for photocatalysis demonstration. A) Schematics illustrating the experimental demonstration of methylene orange (MO) dye degradation under UV light using an AJ‐printed ZnO microlattice. B) Optical images of dye degradation after UV exposure at different times. C) UV–vis absorption kinetics and pseudofirst order rate of MO photodegraded by bulk ZnO (with same weight as microlattice), bulk ZnO (with same volume as the microlattice), a 3D ZnO microlattice fabricated by our technique, and only alumina substrate (control). Note that the equivalent time is calculated based on the assumption where the volume of polluted liquid is equal to the volume of the structure. D) Plot illustrating superior degradation efficiency of the ZnO 3D microlattice when compared to bulk when normalized for projected area.

The unique 3D ceramic microarchitectures fabricated by 3D‐AJP exhibit high surface‐area‐to‐volume ratio due to the lattice structure as well as the surface roughness of the truss members (Figures 5C and S9C,D, Supporting Information). This quality alone will lead to advancements in several technologically important areas. To explore the potential advantages of such structures, we conducted photocatalysis experiments that specifically utilized the active surfaces of ZnO under ultraviolet (UV) light to decompose organic pollutants in aqueous solutions. The schematic in **Figure** **6**A illustrates the setup for this experiment. A sintered ZnO microlattice made by this technique was immersed in methyl orange (MO) solution. The MO solution, along with the microlattice, was then placed under UV irradiation. The photocatalytic performance depends on the surface area of the ZnO, where organic dyes (here, MO) are decomposed by the hydroxyl radicals that act as oxidizing intermediate. The mechanism for this photocatalytic decomposition process is explained as follows

(1) $$\mathrm{ZnO}+hv\;\to \mathrm{ZnO}\left({e_{\mathrm{CB}}}^{-}+{h_{\mathrm{VB}}}^{+}\right)$$

(2)

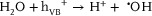



(3)

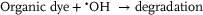




Under the UV irradiation with energy greater than its bandgap (3.2 eV), the photogenerated electrons (e_CB_^−^) and holes (h_VB_^+^) in the valence band are generated on the surface of the ZnO microlattices. The holes then react with water (H_2_O) to form hydroxyl radicals ( ^•^OH) that decompose/degrade organic dyes such as MO by providing a powerful oxidation effect. It is noted that although high surface area enabled surface roughness plays a significant role in this degradation process, the unique open‐cell structure of the microlattice is also expected to help expedite the degradation by facilitating liquid ingress through the entire structure compared to bulk ZnO.

Figure 6B shows a series of optical images of samples under UV irradiation after 0, 24, 48, and 72 h. To better differentiate the results by color, the concentration of MO used here is 100 mg L^−1^, which is significantly higher than the concentration used in other studies from literature.^[^ ^47^ ^]^ Figure 6B shows that the MO dye degraded consistently under UV irradiation as the orange color turned lighter with time.

To isolate the effect of microlattice structure of ZnO on MO degradation, two bulk ZnO cubes that have either similar weight (4.3 mg) or the overall size (which had a weight of 14.2 mg) as that of the microlattice fabricated by our technique (3.5 mg) were also used to degrade MO using UV light under identical conditions. UV–vis spectroscopy was used to compare the results quantitatively, as shown in Figure 6C. Note that *C*_0_ and *C* are the concentrations of MO dye initially and after purification, respectively.

Figure 6C shows that at 20 h, the MO concentration is 37.5%, 57.5%, or 72.8% of initial concentration for the ZnO microlattice, ZnO block of same overall size, and ZnO block of same overall weight, respectively. Clearly, ZnO microlattice fabricated by our technique is the most effective catalyst to degrade MO under UV light. As expected, the control sample with alumina substrate alone did not degrade the MO under UV irradiation.

The first‐order kinetics of MO degradation are also shown in Figure 6C. The equation to quantitatively determine the first‐order model is described as follows

(4) $$\ln\left(\frac{C_{0}}{C}\right)=kt$$

where *k* is the pseudo‐first‐order rate constant, which is a measure of the photodegradation efficiency. The rate constants *k*, calculated using the identical volume equivalent times, are 0.24, 0.33, and 0.73 min^−1^, for the ZnO with the same weight bulk, same volume bulk, and microlattice, respectively. Note that identical volume equivalent time is calculated by converting the volume of the polluted water to the same volume of the printed sample. For instance, in this study, the dimension of the microlattice is around 1.65 mm × 1.65 mm × 0.95 mm ≈2.5 mm^3^. The polluted water used in the photocatalysis experiment is 2.5 mL (2500 mm^3^), we convert it to $\mathrm{time}\;\mathrm{in}\;h\times \frac{60\;\min}{h}\times \frac{2.5}{2500}$ for the equivalent time. This conversion is necessary for normalization of the results and thus can be potentially compared with results from literature.^[^ ^48^ ^]^ The catalytic effect of our ZnO microlattice was thus 2.21× and 3.04× the solid ZnO of comparable size and weight, respectively. We then evaluated the normalized degradation efficiency per unit projected area for all three samples. Note that the degradation efficiency is represented by the change of MO concentration per minute per unit weight under UV irradiation. The projected area normalized degradation efficiency is shown in Figure 6D, where the data from the samples with equivalent weight and volume are combined (the mass normalized degradation efficiency is given in Figure S10 in the Supporting Information). The microlattice shows a 400% improvement in the normalized degradation efficiency compared to bulk ZnO.

To provide a broader context, we benchmarked our ZnO microlattice enabled by 3D‐AJP with other ZnO‐based photocatalysts reported in the literature in addition to comparing them with bulk ZnO. **Table** **3** presents a comparison of the efficiency and equivalent efficiency of various 3D‐printed ZnO structures. The equivalent efficiency is calculated as $\frac{\left(\frac{C_{0}/C}{t}\right)}{\left(\frac{\mathrm{Photocatalyst}\;\mathrm{volume}}{\mathrm{Testing}\;\mathrm{water}\;\mathrm{volume}}\right)}\;$ to ensure a fair comparison across samples. Our results show that the equivalent efficiencies of previously reported structures are below 0.16 min^−1^, whereas our ZnO microlattices achieved a lower efficiency, demonstrating significant potential for in situ water purification applications.

**Table 3:** Comparison of photocatalytic efficiency of different additively manufactured ZnO 3D structures compared to this work (MeBl: methylene blue; MO: methylene orange. Purification efficiency is calculated by $\left(\frac{C_{0}/C}{t}\right)$. Equivalent efficiency is calculated by $\frac{\left(\frac{C_{0}/C}{t}\right)}{\left(\frac{\mathrm{Photocatalyst}\;\mathrm{volume}}{\mathrm{Testing}\;\mathrm{water}\;\mathrm{volume}}\right)}$).

Method	Dye type	Sample size	Polluted water volume	Efficiency [min^−1^]	Equivalent efficiency [min^−1^]	Reference
ZnO on polymer 3D macrostructure	MO	2000 mm^3^	5 mL	0.06	**0.15**	[47b]
ZnO dip‐coated on acrylonitrile butadiene styrene (ABS)+ZnO 3D macrostructure	MeBl	2500 mm^3^	30 mL	0.005–0.013	**0.06–0.16**	[47a]
3D printed polymer containing reduced graphene oxide (rGO) + ZnO	MeBl	6800 mm^3^	100 mL	0.004	**0.06**	[49]
**3D‐AJP (this work)**	MO	2.5 mm^3^	2.5 mL	0.00035	**0.35**	

### 2.5 Application Demonstration: Cancer Biomarker Sensing Platform

**Figure 7:**
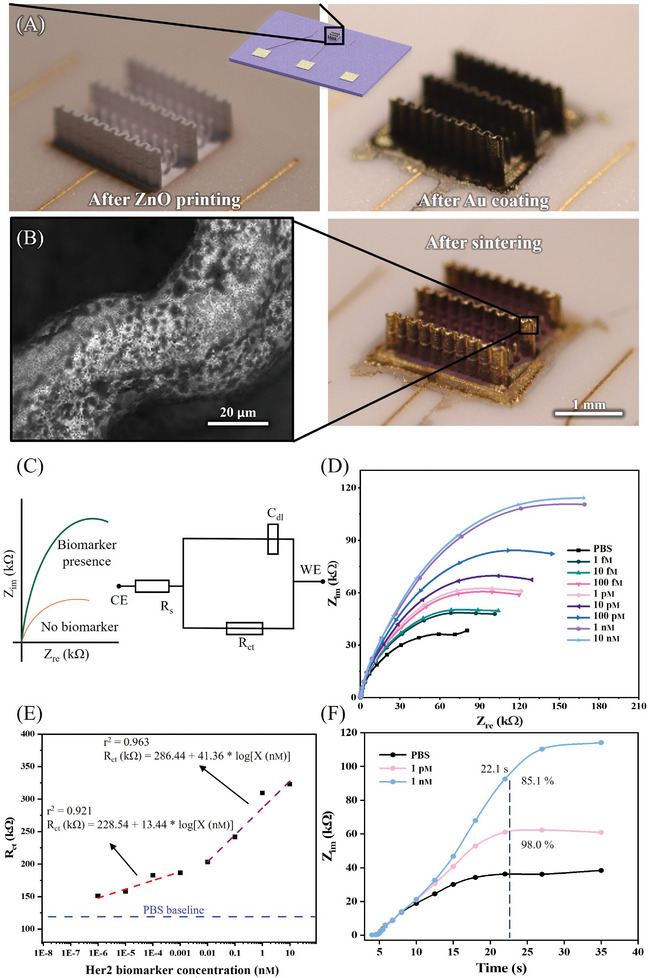
Her2 breast cancer biomarker detection using a sensor with a working electrode (WE) fabricated via our ceramic 3D‐AJP technique. A) Printed WE before (left) and after (right) Au coating, B) SEM (left) and optical (right) images of sintered ZnO wavy wall structure coated with Au. The dark dots indicate Au particles. C) Schematics showing the working principal of our sensor, along with its equivalent circuit diagram. D) Nyquist plots of the ZnO sensor via 3D‐AJP measured by electrochemical impedance spectroscopy (EIS) method without and with the Her2 biomarkers at various concentrations. E) A 2‐range sensitivity plot of ZnO biosensor and F) detection time for Her2 biomarkers at two concentrations.

To explore additional applications of ceramic 3D microarchitectures demonstrated in this paper, we conducted biomolecule sensing experiments using structures enabled by our 3D‐AJP. The specific methods and processes for this demonstration are discussed in detail in the Experimental Section. Oxide nanostructures are widely recognized as highly effective platforms for sensing due to their intrinsic material properties.^[^ ^30a^ ^]^ ZnO nanostructures have demonstrated exceptional sensitivity for biomolecule detection, attributed to their high surface area, biocompatibility, and an ability to electrostatically immobilize proteins on their surfaces.^[^ ^30a,b^ ^]^ The past works in this area, however, have relied only on nanostructures on 2D surfaces.^[^ ^30b,c^ ^]^ Our earlier work demonstrated that sensors with a 3D‐microarchitected electrodes exhibit significantly higher sensitivity compared to a sensor with planar 2D electrodes.^[^ ^50^ ^]^ Lastly, as seen in Figure 5A–D, the 3D‐microarchitected structures we developed in this work have a nanoscale surface texture spread in 3D space, potentially providing a beneficial effect. These observations led us to demonstrate 3D‐microarchitected ZnO made via the 3D‐AJP process as a potential biosensing platform. We detect breast cancer biomarker, namely, human epidermal growth factor receptor 2 (Her2) antigen. The Her2 antigen serves as a breast cancer tumor‐associated antigen, which forms an important biomarker for this disease. Our cell/device consisted of a working electrode (WE), a reference electrode (RE), and a counter electrode (CE). The materials and functionalization of the WE with Her2 biomarkers are described in the “Experimental Section”. **Figure** **7**A shows the optical image of 3D ceramic electrodes made by our 3D‐AJP technique, while Figure 7B shows the microstructure of the working electrode where the brighter parts are sintered ceramic while the darker parts indicate coated Au. Since ZnO is a semiconductor with limited conductivity, the gold coating provides a continuous electron transport pathway. Figure 7C illustrates the schematic of impedance change in the absence and presence of the Her2 biomarkers. An equivalent circuit diagram for the sensor is also illustrated in Figure 7C, where *C*_dl_ is the double layer capacitance on the electrode surface, *R*_ct_ is the charge‐transfer resistance, and *R*_s_ is the electrolyte resistance for the sensor. Impedimetric sensing plot of sensor for Her2 biomarker is shown in Figure 7D. Note that the biomarkers were introduced successively at different concentrations ranging from 1 fm to 10 nm (see the “Experimental Section”). As shown in Figure 7E, the sensor demonstrated a 2‐stage linear response over this concentration range, with a slope of 13.44 ± 2.78 kΩ per 10^−9^ m in the range of 1 × 10^−15^ –1 × 10^−12^ m, and 41.36 ± 5.70 kΩ per 10^−9^ m in the range of 0.01 × 10^−9^ –10 × 10^−9^ m. The limit of detection (LoD) was determined to be 19.3 am (1.93 × 10^−17^ m), based on the calculation using reported literature.^[^ ^51^ ^]^ The calculation is provided in Section S1 (Supporting Information). Note that LoD is the lowest analyte concentration likely to be reliably distinguished from the baseline phosphate‐buffered saline (PBS) signal and at which detection is feasible.^[^ ^51^ ^]^ The detection time was determined to be 22.1 s where the sensor reached 85.1% and 98.0% of their saturation impedance for concentration of 1 pm and 1 nm, respectively, as shown in Figure 7F. This short detection time is consistent with our prior work on 3D‐architected electrodes for biomolecule detection.^[^ ^23^ ^]^ Figure S11 (Supporting Information) shows the deviation of impedance from the mean for each tested concentration (*n* = 5 replicates), showing a low average variance of 1.77%. The results show a clear trend in the increase of circuit impedance indicating the ability of sensing Her2 biomarkers at different concentrations using our 3D‐printed ceramic structures. We conclude that the method developed in this research will lead to ultrarapid and ultrasensitive biosensors.

**Figure 8:**
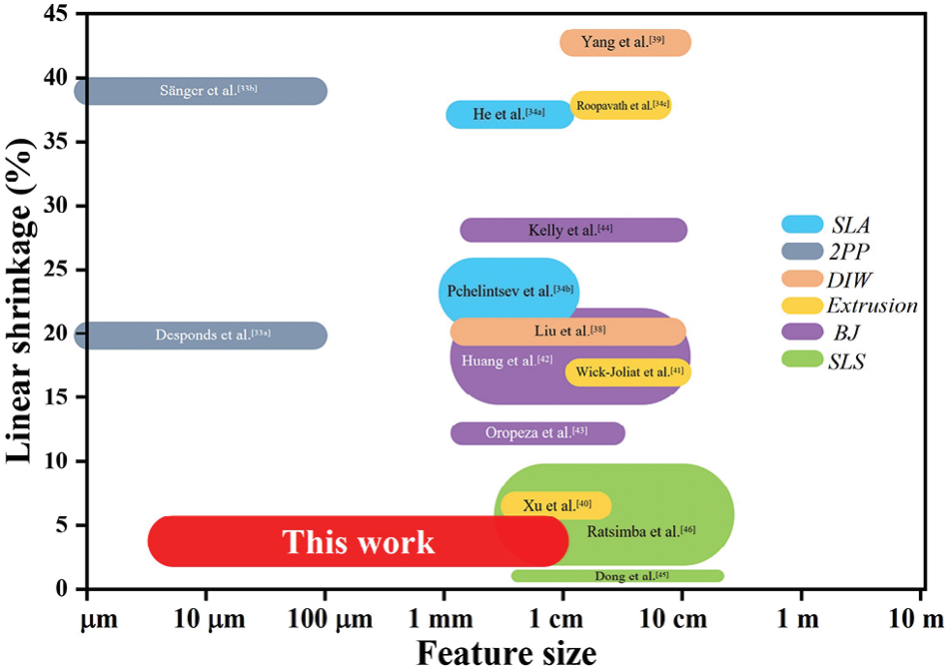
Different ceramic additive manufacturing methods on a map of linear shrinkage versus feature size. The 3D‐microarchitected structures realized by the current work via 3D‐AJP occupies a hitherto unexplored space on this map.

The work presented in this paper has several far‐reaching implications in the fields of ceramic structures and applications. First, the fine structural features of the ceramic microarchitectures demonstrated 3D‐AJP, in combination with ultralow shrinkage, fills a length‐scale gap in the manufacture of ceramics as shown in **Figure** **8**. This combination of features leads to our technique being able to create highly complex and intricate ceramic microarchitectures with high dimensional accuracy (due to low shrinkage), repeatability, and reproducibility. These microarchitectures include lattices, spirals, pillars; opening a large palette of designs that can be realized using our technique. Moreover, the speed of fabrication (e.g., ≈15 min required for the fabrication of a representative microlattices shown in Figure 5) using our technique makes this process scalable, especially when compared to other ceramic AM techniques. Additionally, the on‐demand change in material to integrate multiple ceramic materials in the same structure is a unique aspect of this research. Such a combination of materials can have several applications of their own, including but not limited to the fabrication of high‐entropy ceramics. The novelty and uniqueness of these results are summarized in Tables S2 and S3 (Supporting Information), where the ability to create 3D microscale architectures (for all materials) and the ability to fabricate multimaterial 3D systems are compared across the literature, including that for the current work, respectively.

We also present a manufacturability study of our approach in Figures 2 and 3. These results show that structures can be fabricated by our approach reproducibly and repeatedly. We note, however, that from a modeling perspective, the dynamics of microlattice fabrication including droplet arrival and evaporation would only be captured by multiphysics models that are yet to be developed for the 3D structures. This is especially challenging, as the droplets are ejected at a speed of ≈100 m s^−1^ and travel 2 mm before striking the structure during the micropillar buildup. Our prior work estimates that the evaporation of microdroplets with 5–20 µm diameter would require a few milliseconds to lose most of the solvents.^[^ ^22a^ ^]^ In fact, process modeling is an active area of research in the aerosol Jet 3D printing community. Alternatively, large numbers of experiments would be able to train data‐based models that would also be used to develop a digital twin for the 3D‐AJP process.

Lastly, we speculate about the application space for the ceramic microarchitectures developed in this research. We demonstrate superior catalytic properties of ZnO microlattices compared to bulk material in Figure 6. The high surface‐area‐to‐volume ratio is also highly beneficial in the world of sensing as it greatly increases the sensitivity and sensing time, which is demonstrated by detection of breast cancer biomolecules (Figure 7). The microarchitectures can also be used in microelectronics manufacturing to stiffen electronic packages that would reduce warpage and allow integration of multiple chiplets, enabling 3D heterogeneous integration critical for the artificial intelligence (AI) chips. The lattice can also be used as structural material, especially when composited with infiltrated polymers. These results indicate that the microarchitected ceramics may find applications in structural materials. Lastly, the length scale of the periodicity for the microlattices could lead to applications in IR optics.

## 3 Conclusion

In this paper, we address a significant length‐scale challenge in ceramic fabrication by developing a ceramic 3D aerosol jet printing (3D‐AJP) process. This method enables the creation of complex 3D microarchitected ceramic structures with 20 µm features and minimal shrinkage. We use droplet dynamics to control the assembly of the near‐binder‐free nanoparticles into 3D microarchitectures followed by their sintering, a near‐net‐shape process that does not require any auxiliary support. Complex ceramics such as microspirals, micropillars, microlattices, cellular pyramids, and dumbbells can be fabricated using our manufacturing technique. Larger structures with microscale features but with overall dimensions in centimeters are also demonstrated. Generality of our approach is established with the demonstration of four types of ceramics. Demonstration of a combination of the two ceramic materials in the same architecture as well as a hybrid/mix ceramic architecture establishes 3D multimaterial capability via our approach. We demonstrate that the ZnO microlattices show 400% improvement in photodegradation efficiency per unit projected area compared to bulk ZnO. We also use ceramic 3D‐AJP to construct a 3D ZnO‐based biosensor and show that it can rapidly (in 20–30 s) detect Her2 antigen, an important biomarker for human breast cancer at a very low limit of detection of 0.0193 fm, demonstrating the breadth of applications enabled by our work. The ceramic 3D‐AJP technique we developed thus opens new frontiers in areas that include, but are not limited to, microelectronics, catalysis, structural materials, biomolecule sensing, tissue regeneration, thermal and environmental barriers, and filtration.

## 4 Experimental Section

### ZnO, ZrO_2_, TiO_2_, and Al_2_O_3_ Nanoparticle Inks

A commercial ZnO nanoparticle ink (item 721077, CAS #1314‐13‐2, Sigma‐Aldrich, St. Louis, MO, USA) with a density of 1.7 g cm^−3^, an average particle size of 40 nm (TEM image shown in Figure S1B in the Supporting Information) with the largest particle of <100 nm, and a particle loading of 20 wt% in H_2_O was utilized to form the 3D microarchitectures. Three ink concentrations were used to obtain the critical angle for 3D structures shown in Figure 2: original with no dilution (20 wt%), mixed with deionized (DI) water at ratios of 2:1 (≈13 wt%), and 2:3 (≈8 wt%). A commercial ZrO_2_ nanoparticle dispersion (item #643025, CAS #1314‐23‐4, Sigma‐Aldrich, St. Louis, MO, USA) was used in this work and had a density of 1.3 g cm^−3^, a particle size of <100 nm, and a particle loading of 10 wt% in H_2_O. This ink was used without modifications. A commercial TiO_2_ nanoparticle ink (item # 700347, CAS #13463‐67‐7, Sigma‐Aldrich, St. Louis, MO, USA) used in this work and had a particle size of <150 nm and a particle loading of 40 wt% in H₂O. This ink was modified by mixing it in DI water at a volume ratio of 1:1 prior to printing. A commercial Al_2_O_3_ nanoparticle ink (item # 642991, CAS #1344‐28‐1, Sigma‐Aldrich, St. Louis, MO, USA) with particle sizes in the range of 30–60 nm and a particle loading of 20 wt% in H₂O was used for the demonstration of hybrid ceramics.

### Hybrid Ceramic Nanoparticle Ink

The hybrid ink to print the structure shown in Figure 4F was prepared by mixing ZnO, TiO_2_, and Al_2_O_3_ nanoparticles into a dispersion. Direct mixing of the ink led to sedimentation due to proprietary additive from the ink supplier. This issue was addressed in the following manner. Each nanoparticle dispersion was centrifuged at 4000 rpm for 10 min, and the solvent was replaced with DI water. This process was repeated five times. The nanoparticles were then dried in an oven (Neytech Vulcan furnace, Model 3–550, Degussa‐Ney Dental Inc., Bloomfield, CT, USA) at 70 °C for 1 h. After drying, the particles were mixed in a 1:1:1 weight ratio and redispersed in DI water with a final weight percent of 20%. Probe sonication was used for sediment prevention and to ensure uniformity of the ink. After sonication, 1.5 mL of the ink was transferred to the ultrasonic atomizer of the AJ printer.

### Metal Inks

The commercially available Ag nanoparticle ink (PRELECT TPS 30, Clariant, Muttenz, Switzerland) and Au nanoparticle ink (UTDAu40, UT Dots Inc., Champaign, IL, USA) were used to compare the percentage of binder/additive content in Figure S2 (Supporting Information). The Ag nanoparticle ink had a density of 1.75 g cm^−3^, a viscosity of about 1.5 cP, a particle size of 30–50 nm, and a particle loading of 40 ± 2 wt%. The Au nanoparticle ink had a viscosity of about 3 cP, a particle size of 2–5 nm, and a particle loading of around 40 wt%.

### 3D‐AJP Process for ZnO, ZrO_2_, TiO_2_, and Al_2_O_3_ Nanoparticles

The 2D and 3D structures were fabricated using an AJ 3D printer (AJ300, Optomec Inc, Albuquerque, NM, USA), which is a microscale AM method. AJ printing is a continuous material jetting process that utilizes transformation of the inks into a mist of aerosol droplets (each droplet containing nanoparticles of functional material) to be deposited at a desired location. In the printing process, ZnO, ZrO_2_, TiO_2_, and the hybrid nanoparticle ink were atomized by ultrasonic energy and carried by N_2_ gas to the deposition head in the form of an aerosol mist. The aerosol droplets were then focused by a sheath gas (also N_2_) through a ceramic nozzle (150 µm in diameter) onto a substrate. The standoff distance between nozzle tip and substrate (alumina substrate with 96% purity, ALN‐101005S1, MTI Corp, Richmond, CA, USA) was initially kept at about 3 mm and would be adjusted during printing. Commercial software AutoCAD (Autodesk, San Rafael, CA, USA) was used to generate the drawings for geometries of both 2D and 3D architectures. The generated drawings were exported into “prg” files that could be read by the AJ printer using Autolisp add‐on provided by Optomec Inc. During printing, the platen was kept at a constant temperature ranging from room temperature to 100 °C for different demonstrations as specified in the paper. The printed green structures were thermally sintered in a programmable oven (Neytech Vulcan furnace, Model 3–550, Degussa‐Ney Dental Inc., Bloomfield, CT, USA) at 950 °C for 6 h.

### Characterization

Imaging was done using a scanning electron microscope (FEI Quanta 600 FE‐SEM, FEI Company, Hillsboro, OR, USA). To ensure better image qualities, the samples, both sintered and unsintered, were sputter‐coated with 5 nm of Au before transferring to SEM chamber. The energy‐dispersive spectroscopy (EDX or EDS) analysis was performed on printed bimaterial lattice using an attachment to the SEM equipment (EDS, Oxford Inca, Oxford Instruments, UK). Focused ion beam (FEI Nova Nanolab 600 Dual Beam FIB, FEI Company, Hillsboro, OR, USA) was used to section a truss member of a printed and sintered ZnO micropillar (as shown in Figure 3C and in Figure S4B in the Supporting Information). A 2 µm thick Pt layer was deposited to protect the surface prior the FIB cut. TGA of as‐received nanoparticle inks used in this work was performed (Q50, TA Instruments, New Castle, DE, USA). The ink was heated in an air environment from 25 to 800 °C at a ramp rate of 10 °C min^−1^.

The XRD study was carried out on the micropillars. The XRD measurement in Figure S4 (Supporting Information) was taken at room temperature with a Malvern PANalytical Empyrean X‐ray diffractometer (PANalytical Inc., EA Almelo, Lelyweg, Netherlands). The XRD used Cu Kα radiation (*λ* = 1.5406 Å) at 45 kV and 40 mA. Scans were taken in Bragg–Brentano geometry with a 0.05° step size and a 10 s dwell. For all the scan results, lattice parameters and phase ratios were least‐square fit using Rietveld refinement with HighScore Plus software (PANalytical, EA Almelo, Lelyweg, The Netherlands). Optical images were taken using an optical microscope (Canon EOS Ti7 Rebel, Canon Corp., Tokyo, Japan). Porosity was measured using ImageJ software (National Institutes of Health, Bethesda, MD, USA). SEM images were converted to 8‐bit grayscale to facilitate thresholding. Then a local threshold method (Sauvola, v1.11.0) was used to differentiate the pores (darker) with the solid (lighter). After the analysis was finished, the threshold adjustment tool could reveal the planner porosity and the same procedures were performed five times on different locations in each SEM image, and the plots were generated using OriginLab Pro (OriginLab Inc., Northampton, MA, USA).

### Photocatalysis Studies

The ZnO microlattice used in this study had a dimension of 1.65 mm × 1.65 mm × 0.92 mm thus a total volume of 2.59 mm^3^. Two commercially available bulk ZnO samples were used to compare the degradation rate, representing the same volume (14.8 mg) and same weight (3.45 mg) as the microlattice. Alumina substrates were also used for control purposes. For UV–vis measurements, it is recommended to use at least 2.5 mL of aqueous solution, which is 966 times volume compared to the microlattice. Thus, the MO dye used for UV–vis measurements was diluted to a concentration of 4 mg L^−1^ to expedite the photocatalysis process. The aqueous solutions containing MO dye together with all the samples were put inside containers under an UV lamp of 350 nm shining with normal incidence. The solution before and during decontamination were tested using a UV–vis spectrometer (Lambda 900, Perkin‐Elmer, Waltham, MA) at time points of 0, 1, 3, 7, 13, and 20 h.

### Functionalization of Biosensor and Her2 Biomarker Detection

A commercial Au nanoparticle ink (UTDAu40, UT Dots Inc., Champaign, IL, USA) with 40 ± 2 wt% particle loading, 2–4 nm particle size, and a viscosity of 3 cP, was used to fabricate the base (a 2D film) of the RE, WE, and CE via AJP. The ink container was rolled at 70 rpm for 30 min before using the ink in the AJ printer. Sintering of the electrodes was conducted at 400 °C for 3 h. After sintering, 3D ZnO wavy planes and micropillars were printed on top of the gold film (Figure 7A) followed by a sintering at 950 °C for 6 h. Au ink (20 µL in volume) was then manually drop‐casted onto the printed WE to form a thin layer of Au coating followed by a final sintering at 400 °C for 6 h. The gold provides a path for electron conduction for the sensor. The RE was coated with commercial Ag/AgCl ink (Ercon, Inc. Wareham, MA, USA) via drop‐casting assisted by a Kapton shadow mask.

ZnO has an IEP of ≈9.5,^[^ ^30a^ ^]^ while that for the Her2 biomarkers is in the range of 4–7.^[^ ^52^ ^]^ Under these conditions, ZnO is positively charged, and the Her2 biomarker is negatively charged, and is known to facilitate electrostatic immobilization of the biomolecule on ZnO.^[^ ^30b^ ^]^ To further enhance the antibody loading, the WE was functionalized with 11‐mercaptoundecanoic acid and then with Her2 antibodies (Biovision, Abcam, Waltham, Boston, MA, USA) using the 1‐Ethyl‐r‐(3‐dimethylaminopropyl)carbodiimide (EDC) chemistry, following a previously developed procedure.^[^ ^23^ ^]^ The electrode characterization and recording of electrochemical signals were done using VersaSTAT 4 Potentiostat Galvanostat (Ametek Inc., Berwyn, PA, USA). VersaStudio software from the same company was used to analyze the data to obtain the charge‐transfer resistance values. A PDMS housing (following the same procedure as in Ali et al.^[^ ^23^ ^]^) was used for the electrochemistry experiment. Her2 antigen powder (Biovision, Abcam, Waltham, MA, USA), to be detected, were dissolved in a mix of PBS and 5 mm of ferro/ferricyanide to form a 10 nm solution. This solution was sequentially diluted to create concentrations ranging from 1 fm to 1 nm. Control experiment was carried out with PBS with ferro/ferricyanide without the Her2 biomarker. The experiment was conducted sequentially from the lowest concentrations, with each concentration being tested five times. The raw data are provided in the Supporting Information. After each concentration, the device was rinsed three times with PBS before the next test.

## Conflict of Interest

The authors declare no conflict of interest.

## Author Contributions

R.P. came up with the concept and directed the research. C.H. built the devices and carried out all the fabrication and wrote the first draft of the paper. S.J. contributed to SEM, FIB, ink characterization, TGA, in addition to printing. B.Y. helped with ink optimization, UV–vis, and biomarker test. R.P. and C.H. co‐edited the manuscript. All authors commented on the results.

## Supporting information



Supporting Information

Supplemental Movie 1

Supplemental Table S1

## Data Availability

The data that support the findings of this study are available from the corresponding author upon reasonable request.
