# Supplementary material for: 3D‐AJP: Fabrication of Advanced Microarchitected Multimaterial Ceramic Structures via Binder‐Free and Auxiliary‐Free Aerosol Jet 3D Nanoprinting
Source: Adv Sci (Weinh). 2025 Feb 7;12(15):2405334. doi: 10.1002/advs.202405334 (PMC12005755; doi:10.1002/advs.202405334)
Supplement: Supplementary file 1 — Supporting Information [file ADVS-12-2405334-s001.pdf]

ADVANCED SCIENCE
Open Access

Supporting Information

for *Adv. Sci.*, DOI 10.1002/advs.202405334

3D-AJP: Fabrication of Advanced Microarchitected Multimaterial Ceramic Structures via Binder-Free and Auxiliary-Free Aerosol Jet 3D Nanoprinting

*Chunshan Hu, Sanjida Jahan, Bin Yuan and Rahul Panat**

Supplementary Information for

3D-AJP: Fabrication of Advanced Microarchitected Multi-Material Ceramic Structures via Binder-Free and Auxiliary-Free Aerosol Jet 3D Nanoprinting

Chunshan Hu[1], Sanjida Jahan[1], Bin Yuan[1], and Rahul Panat[1,2,3,*]

[1]Department of Mechanical Engineering, Carnegie Mellon University, Pittsburgh, PA 15213 USA

[2]Robotics Institute, Carnegie Mellon University, Pittsburgh, PA 15213 USA

[3]Manufacturing Futures Institute, Carnegie Mellon University, Pittsburgh, PA 15213 USA

* Corresponding author. Email: rpanat@andrew.cmu.edu

Section S1
Table S1
Table S2
Table S3
Supplementary Figure S1
Supplementary Figure S2
Supplementary Figure S3
Supplementary Figure S4
Supplementary Figure S5
Supplementary Figure S6
Supplementary Figure S7
Supplementary Figure S8
Supplementary Figure S9
Supplementary Figure S10
Supplementary Figure S11
Supplementary Movie S1: Printing of a ZnO 3D microlattice structure via 3D-AJP. The lattice printing for the video was carried out at a slower platen speed for clarity and took about 35 minutes of printing time. At the standard platen speed, the time to print a lattice in Fig. 3D would be about 15 minutes.

Section S1: Limit of detection calculation for the Her2 biosensor.

The limit-of-detection (LoD) was calculated using a method described by Armbruster et al. [1]. The calculation involved evaluating limit-of-blank (LoB) and then LoD in terms of concentration [2] using,

LoB = Mean of signal (blank sample) + 1.645 × (Std dev of blank sample) (1)

Limit-of-detection of the signal (Y_{LoD}) = LoB + 1.645 × (Std dev of target at low concentration) (2)

LoD = (Y_{LoD} - *c*)/slope of the sensor calibration. (3)

Note that LoB is the highest apparent analyte concentration expected to be found when replicates of a blank sample containing no analyte are tested, while LoD is the lowest analyte concentration likely to be reliably distinguished from the LoB and at which detection is feasible [1].

Here, '*c*' is the intercept of the calibration curve of the sensor (Figure 7E). For ZnO-based sensor, the mean of the blank signal and standard deviation (Std dev) are 119.15 kΩ and 1.58 kΩ (n, replicate = 5). From Eq. (1), this gives LoB = 121.74 kΩ. Mean signal at the lowest concentration (1 fM of biomarker) is 150.90 kΩ, with a standard deviation of 1.89 kΩ (n, replicate = 5). This gives Y_{LoD} = 124.85 kΩ as per Eq. (2). The sensor calibration equation (Figure 7E) is R_{ct} (kΩ) = 228.54 + 13.44 × Log [X (nM)]. This gives *c* = 228.54 kΩ and slope of the sensor calibration curve as 13.44 kΩ. Plugging into Eq. (3), we can have the log(X) = (124.85 – 228.54)/13.44 = -7.72, which gives X = 1.93×10^{-8} nM = 0.0193 fM = 19.3 aM. Thus, the LoD of our ZnO-based sensor is 19.3 aM.

Table S1. Comparison of the dimensions of tilted 3D ZnO micropillars printed at different temperatures using 3D-AJP (Fig. 2A-C).

Printing temperature	Room temperature	50 °C
Diameter (μm)	95.8	74.9
Radius (μm)	47.9	37.4
Length (μm)	666.9	963.3
Volume (μm^3)	4.81×10^6	4.24×10^6
Volume difference	11.8%	

Table S2: Comparison of three-dimensional microarchitected materials fabricated by different manufacturing techniques.

AM method	Material	Process	Minimum feature	Speed	Multi-material capability	Application demonstration	Ref
2PP	Polymer, Ceramic (w/hydrogel infusion)	Complex, multi-step	~ 20 nm	Slow	Limited - Stacked	Biomedical Optoelectronics Micro-fluidics Micro-robotics	[3-6]
EHD jet printing	Polymer Metal Ceramics Biomaterials	Complex, multi-step	~ 200 nm	Slow	Limited - Stacked	Electronics Biomedical Optics	[7, 8]
AJP	Metal (our prior work) ITO (pillars)	Simple	~ 10 μm	Fast	NA	Electronics Photosynthesis	[9-12]
**AJP (this work)**	**Ceramics - Complex structures of $ZnO + Al_2O_3 + ZrO_2 + TiO_2$**	**Simple**	**~ 10 μm**	**Fast**	**Excellent**	**Biosensing Insulation Photocatalyst**	

2PP: 2-photon polymerization
μSLA: micro- stereolithography
FDM: fused deposition modeling.
DIW: direct ink writing.
SLS: selective laser sintering.
IJP: inkjet printing.
EHD jet printing: electrohydrodynamic jet printing.
AJP: aerosol jet printing

Table S3: Comparison of multi-material additive manufacturing methods.

Material category	Material	AM process	Smallest feature	Relative *z*-build speed	Process steps	Complexity	Multi-mat Type	Ref
Polymer-Polymer	PLA+ Nylon	FDM	~ 300 µm	Fast	1	High	Stacked	[13]
Polymer-Polymer	PETA+ PEGDA	2PP	~1 µm	Slow	3	High	Stacked	[14]
Metal-Metal	316L+ CuSn10	LPBF	~ 200 µm	Medium	1	High	Limited - Stacked	[15]
Metal-Metal	AlSi+ AlCuMg Si	SLM	~ 500 µm	Fast	1	Low	Limited - Stacked	[16]
Ceramic-Ceramic	Silica sand+ Zircon sand	SLS	~ 500 µm	Fast	1	Low	Limited - Stacked	[17]
Ceramic-Ceramic	B_4C +SiC	DIW	~ 1 mm	Fast	2	Low	Stacked + Hybrid	[18]
Metal-Ceramic	Steel + Zirconia-Alumina	SLM	~ 300 µm	Fast	1	Low	Limited - Stacked	[19]
Metal-Ceramic	Cu + Al_2O_3	LOM	~ 1 mm	N/A	3	Low	Limited - Stacked	[20]
**Ceramic-Ceramic**	**ZnO + Al_2O_3 + ZrO_2**	**AJP (This work)**	**20 µm**	**N/A**	**2**	**High**	**Stacked +Hybrid**	

FDM: fused deposition modeling. It takes roughly **1 - 2 minutes** for a microlattice with 1 mm × 1 mm × 1 mm in size to be built
LPBF: laser powder bed fusion. 1 mm × 1 mm × 1 mm too small to build.
SLM: selective laser melting. 1 mm × 1 mm × 1 mm too small to build.
SLS: selective laser sintering. 1 mm × 1 mm × 1 mm too small to build.
DIW: direct ink writing. It takes roughly **1 - 2 minutes** for a microlattice with 1 mm × 1 mm × 1 mm in size to be built
LOM: laminated object manufacturing.
2PP: 2-photon polymerization. It takes roughly **10 hours** for a microlattice with 1 mm × 1 mm × 1 mm in size to be built
3D-AJP: 3D aerosol j printing. It takes roughly **15 minutes** for a microlattice with 1 mm × 1 mm × 1 mm in size to be built

Figure S1. Ceramic (ZnO) ink characterization and 2D printability study. (A) TGA of ZnO nanoparticle ink showing low weight percent of binders/additives. (B) TEM of the ZnO ink showing the nanoparticle size and morphology. (C) SEM images of AJ printed 2D traces under different printing parameters at room temperature. The green zone marks the optimized printing conditions. Scale bar: 25 μm. (D) SEM of a representative printed 2D trace showing smooth edge and uniform width on alumina substrate under optimum printing parameters. (E) Diameter of droplets (after the spread on impact with the substrate) on both alumina and silicon wafer substrates deposited at different temperatures.

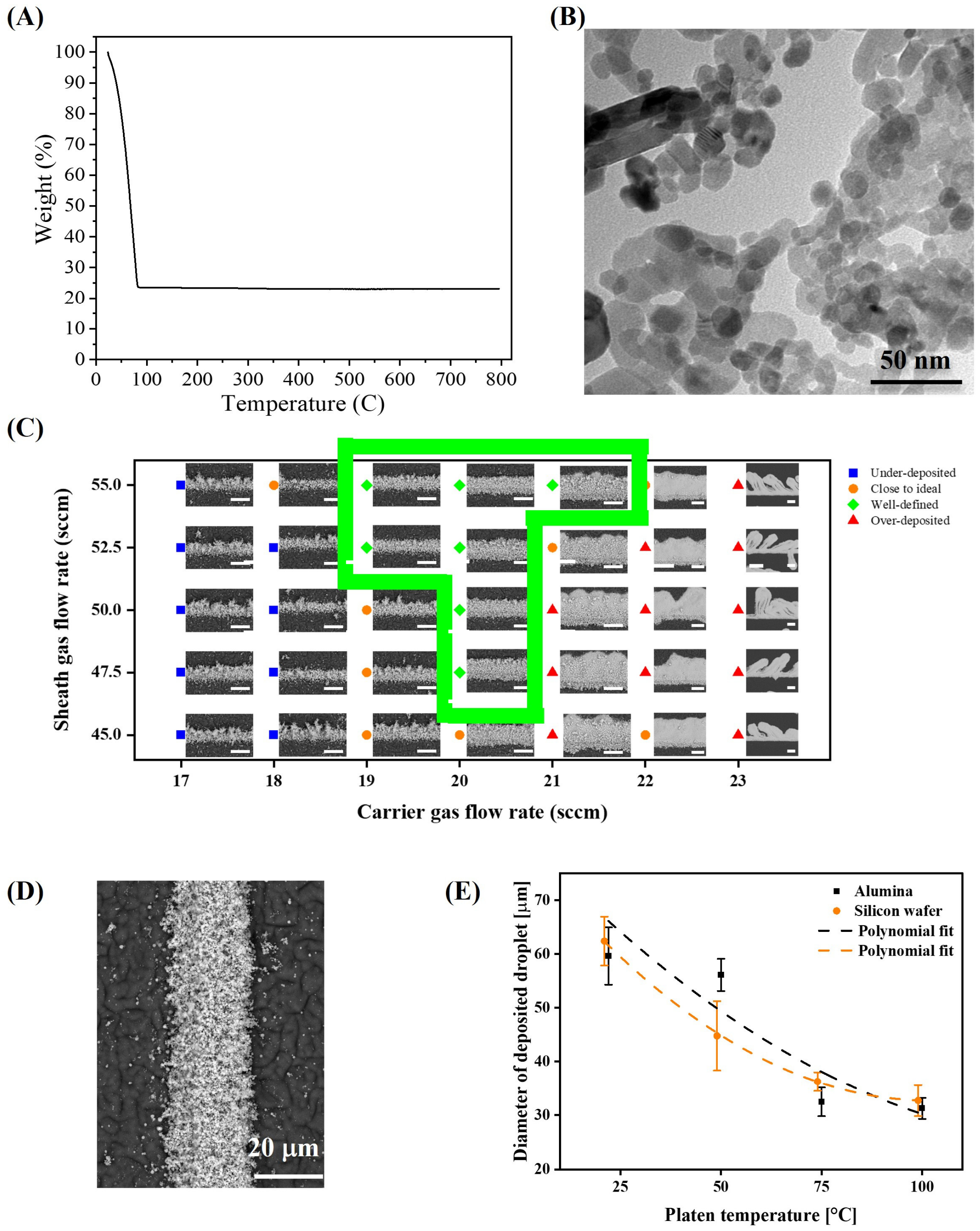

Figure S2. TGA of commercially available (A) silver and (B) gold inks for AJ printing.

(A)

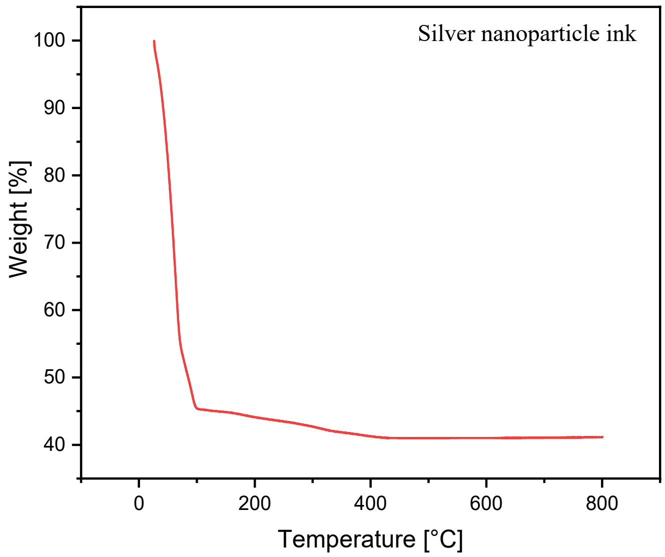

(B)

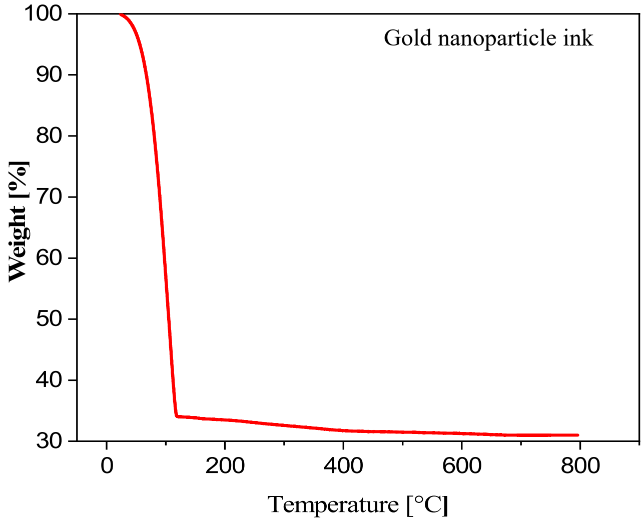

Figure S3. Volume shrinkage of ZnO ceramic micro pillars shown in Fig. 3B.

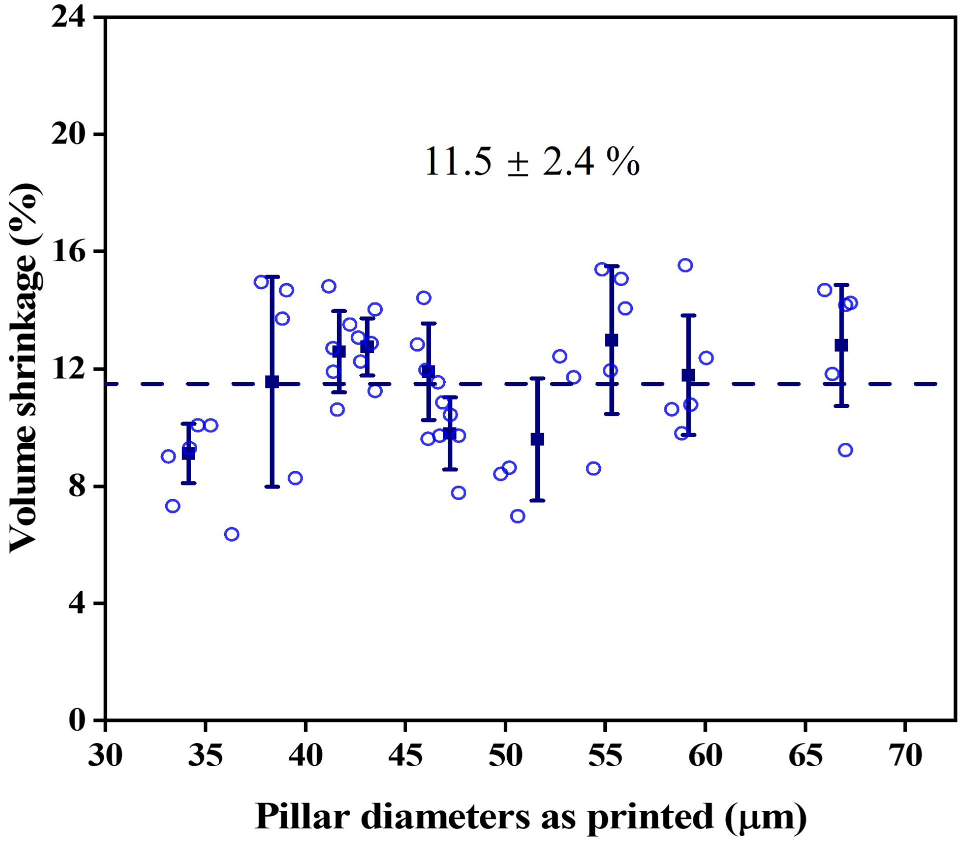

Figure S4. FIB cross-section and porosity measurement. (A) SEM image of the cross-section of one struct of the microlattice before sintering via FIB sectioning. Dense packing of particles is observed with a porosity of 9.12%. (B) Porosity measurement for the sintered ZnO truss cross section shown in Fig. 3C (porosity of 8.62%).

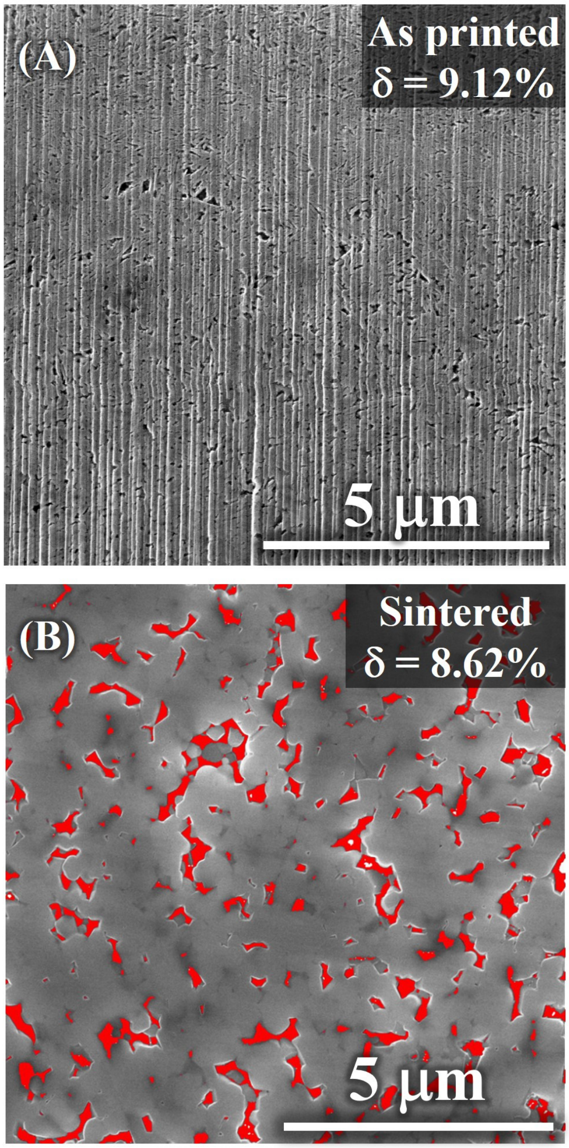

Figure S5: XRD plot of sintered ZnO microlattice.

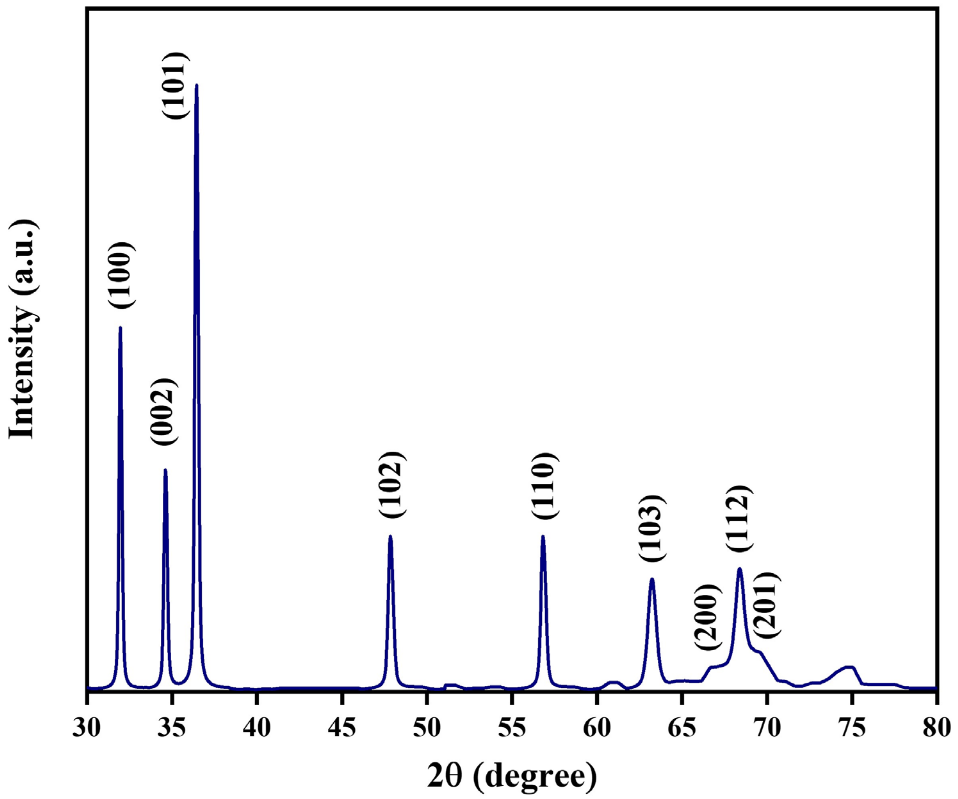

Figure S6. Schematics showing the design and fabrication process of ceramic micro-lattices. (A) Schematic of a 3×3×5 lattice rendered in SolidWorks, (B) AutoCAD drawing representing one layer of printing program, with the zoomed-in image showing the details of the printing of one truss member, and (C) comparison between the fabricated sample and the 3D model.

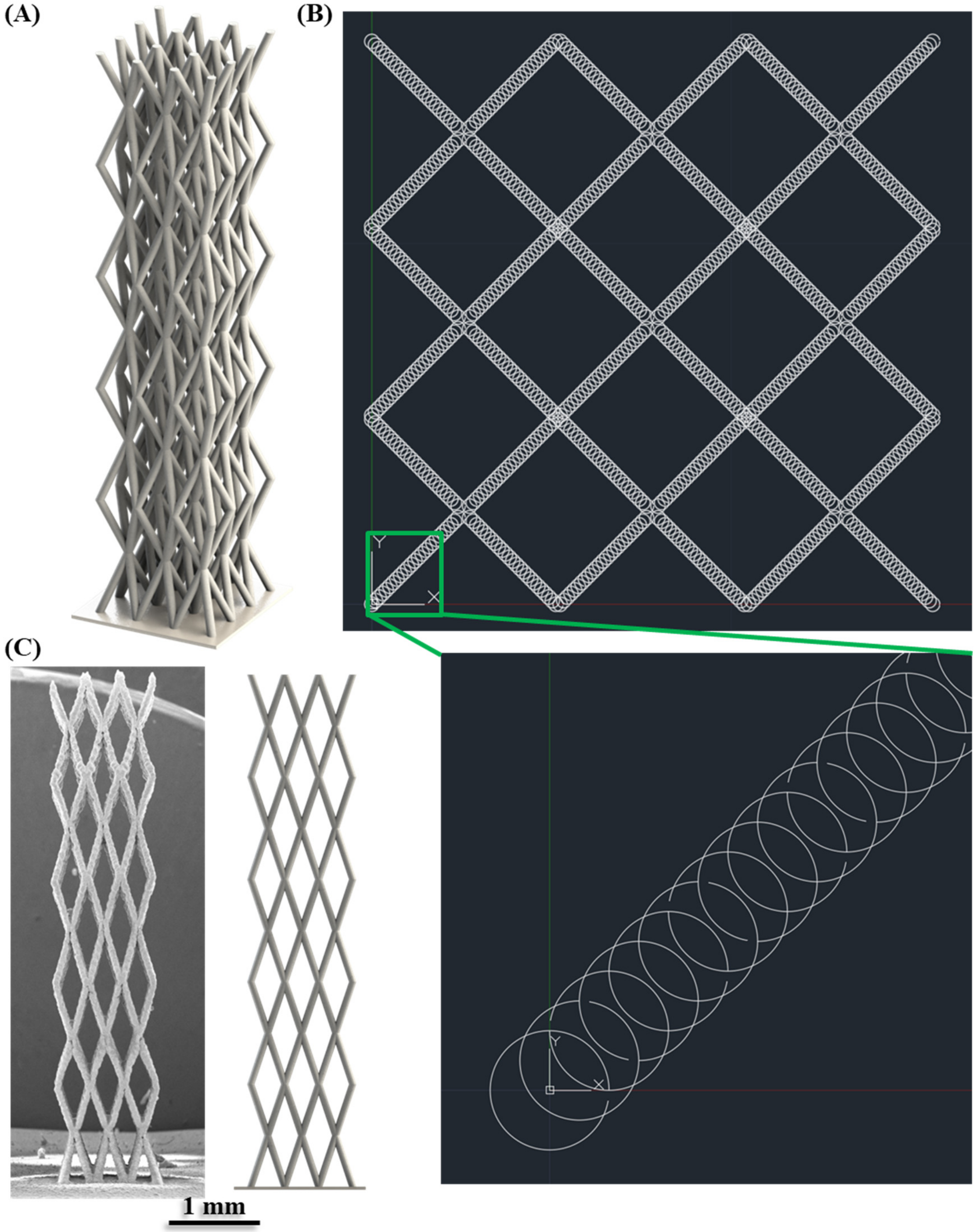

Figure S7. ZnO 3D micro-lattices via the fabrication approach described in the paper. (A) A representative 3×3×5 microlattice fabricated by our approach and zoomed-in SEMs showing the top view and struts and joints. (B) Truss diameters showing variation within ± 6% of the mean diameter.

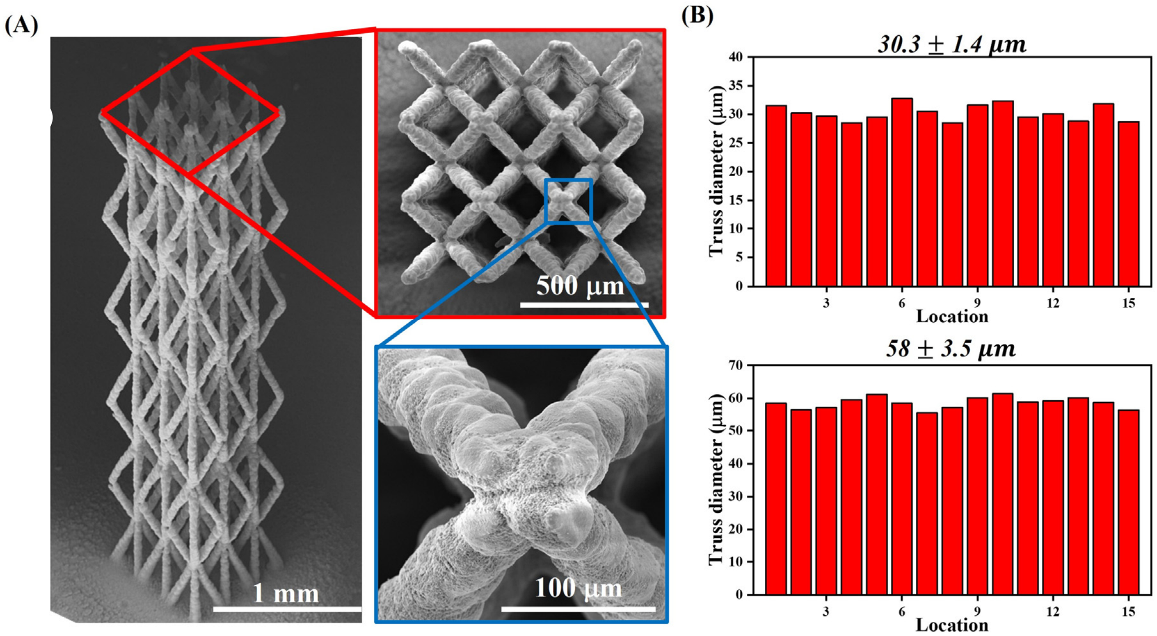

Figure S8. Fabrication of ceramic 3D microarchitectures via the approach described in the paper. Dumb-bell shaped microarchitecture of ZnO: CAD design (left) and as fabricated structure (right). Scale bar is 500 μm.

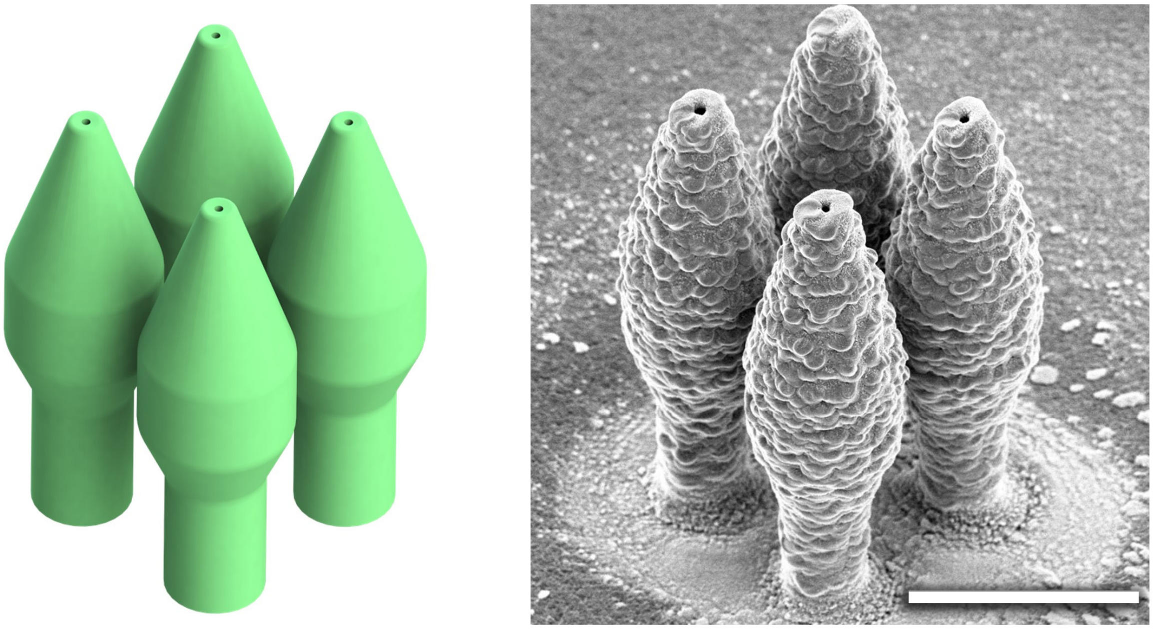

Figure S9. Additional hierarchically porous microarchitectures created by our technique with pore/feature sizes spanning over five orders of magnitude in length scale. (A) SEM of a 3×3×5 microlattice. (B) SEM of truss members of the lattices having thicknesses in the range of 30 – 80 μm. Lattice periodicity of 100 - 350 μm is also observed. (C) SEM of pores on the surface of the struts, and (D) porosity revealed by FIB cross-sectioning with pore sizes of hundreds of nanometers.

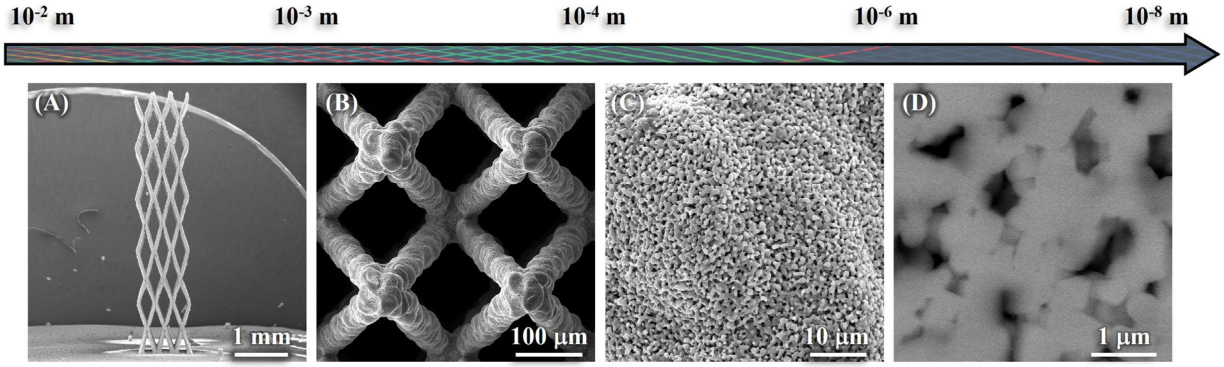

Figure S10. Degradation efficiency of bulk ZnO and ZnO NP-based micro-lattice per unit mass under UV radiation.

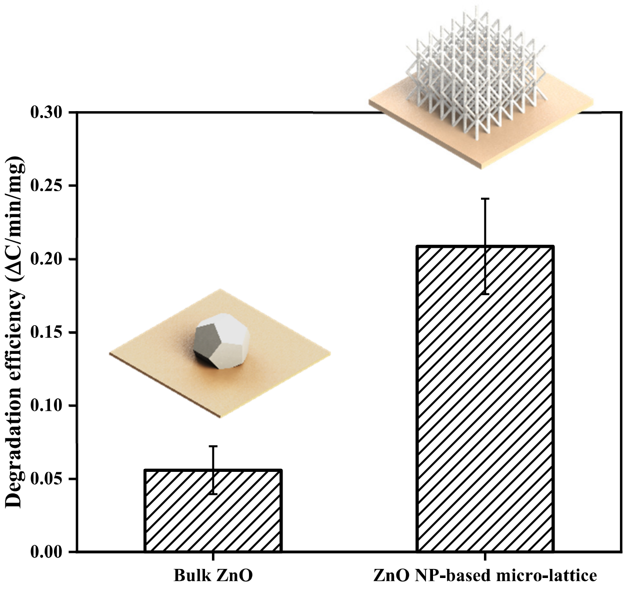

Figure S11. Her2 antigen detection variation. (A) Nyquist plots of the ZnO sensor via 3D-AJP measured using the EIS method with Her2 antigen at 1 pM for five repeated tests. (B) R_{ct} variation of ZnO sensor at various concentrations. The signal variance for each concentration is determined by summing the squares of deviation from the mean, dividing by number of data points (n = 5). The average of such variances for all the samples is 1.77% with a standard deviation of 0.79%.

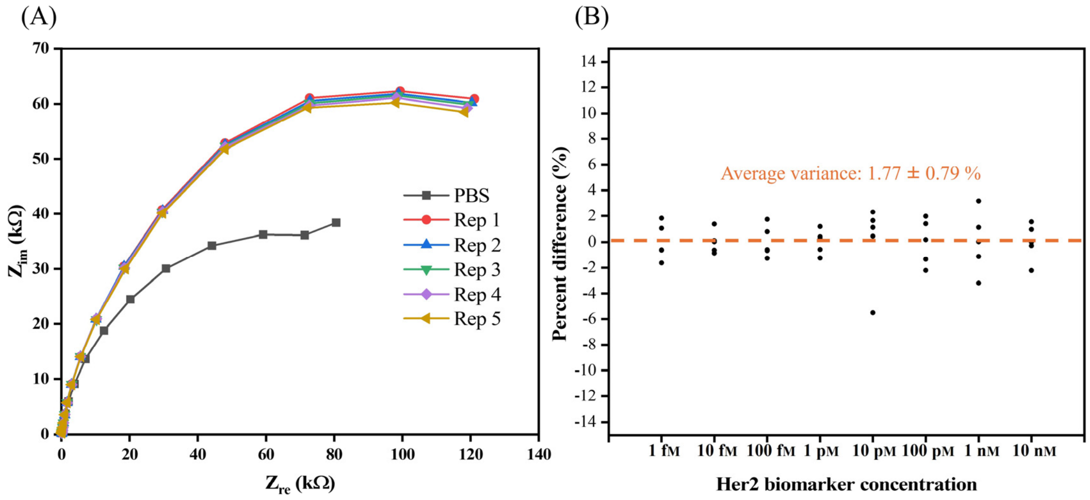

References:

1. Armbruster, D.A. and T. Pry, *Limit of blank, limit of detection and limit of quantitation.* The clinical biochemist reviews, 2008. **29**(Suppl 1): p. S49.
2. Lavín, Á., et al., *On the determination of uncertainty and limit of detection in label-free biosensors.* Sensors, 2018. **18**(7): p. 2038.
3. Obata, K., et al., *High-aspect 3D two-photon polymerization structuring with widened objective working range (WOW-2PP).* Light: Science & Applications, 2013. **2**(12): p. e116-e116.
4. Wu, S., J. Serbin, and M. Gu, *Two-photon polymerisation for three-dimensional micro-fabrication.* Journal of Photochemistry and Photobiology A: Chemistry, 2006. **181**(1): p. 1-11.
5. Song, J., et al., *From simple to architecturally complex hydrogel scaffolds for cell and tissue engineering applications: Opportunities presented by two-photon polymerization.* Advanced healthcare materials, 2020. **9**(1): p. 1901217.
6. Li, M., et al., *Low-temperature 3D printing of transparent silica glass microstructures.* Science Advances, 2023. **9**(40): p. eadi2958.
7. Meng, Z., et al., *Micro/nanoscale electrohydrodynamic printing for functional metallic structures.* Materials Today Nano, 2022. **20**: p. 100254.
8. Reizabal, A., et al., *Electrohydrodynamic 3D printing of aqueous solutions.* Small, 2023. **19**(7): p. 2205255.
9. Ali, M.A., et al., *Sensing of COVID-19 antibodies in seconds via aerosol jet nanoprinted reduced-graphene-oxide-coated 3D electrodes.* Advanced Materials, 2021. **33**(7): p. 2006647.
10. Saleh, M.S., C. Hu, and R. Panat, *Three-dimensional microarchitected materials and devices using nanoparticle assembly by pointwise spatial printing.* Science advances, 2017. **3**(3): p. e1601986.
11. Chen, X., et al., *3D-printed hierarchical pillar array electrodes for high-performance semi-artificial photosynthesis.* Nature Materials, 2022. **21**(7): p. 811-818.
12. Smith, B.N., et al., *Aerosol Jet Printing Conductive 3D Microstructures from Graphene Without Post-Processing.* Small, 2024. **20**(12): p. 2305170.
13. Sarvestani, H.Y., et al., *Engineered bi-material lattices with thermo-mechanical programmability.* Composite Structures, 2021. **263**: p. 113705.
14. Hu, Q., et al., *The influence of printing parameters on multi-material two-photon polymerisation based micro additive manufacturing.* Additive Manufacturing, 2022. **51**: p. 102575.
15. Chen, K., et al., *Selective laser melting 316L/CuSn10 multi-materials: Processing optimization, interfacial characterization and mechanical property.* Journal of Materials Processing Technology, 2020. **283**: p. 116701.
16. Desponds, A., et al., *3D Printing and Pyrolysis of Optical ZrO2 Nanostructures by Two-Photon Lithography: Reduced Shrinkage and Crystallization Mediated by Nanoparticles Seeds.* Small, 2021. **17**(42): p. 2102486.
17. Shan, Z.-d., et al., *Coating process of multi-material composite sand mold 3D printing.* China foundry, 2017. **14**(6): p. 498-505.
18. Pelz, J.S., et al., *Multi-material additive manufacturing of functionally graded carbide ceramics via active, in-line mixing.* Additive Manufacturing, 2021. **37**: p. 101647.
19. Koopmann, J., J. Voigt, and T. Niendorf, *Additive manufacturing of a steel–ceramic multi-material by selective laser melting.* Metallurgical and Materials Transactions B, 2019. **50**: p. 1042-1051.
20. Pfeiffer, S., et al., *Al2O3/Cu-O composites fabricated by pressureless infiltration of paper-derived Al2O3 porous preforms.* Ceramics International, 2018. **44**(17): p. 20835-20840.
